# New fossil discoveries illustrate the diversity of past terrestrial ecosystems in New Caledonia

**DOI:** 10.1038/s41598-021-97938-5

**Published:** 2021-09-15

**Authors:** Romain Garrouste, Jérôme Munzinger, Andrew Leslie, Jessica Fisher, Nicolas Folcher, Emma Locatelli, Wyndy Foy, Thibault Chaillon, David J. Cantrill, Pierre Maurizot, Dominique Cluzel, Porter P. Lowry, Peter Crane, Jean-Jacques Bahain, Pierre Voinchet, Hervé Jourdan, Philippe Grandcolas, André Nel

**Affiliations:** 1 Institut de Systématique, Evolution, Biodiversité, Muséum National d’histoire Naturelle, CNRS, SU, EPHE, UA, 45 rue Buffon, CP50, 75005 Paris, France; 2 AMAP, Université Montpellier, IRD, CIRAD, CNRS, INRAE, 34000 Montpellier, France; 3 Department of Geological Sciences, Stanford University, 450 Jane Stanford Way450 Jane Stanford Way, Building 320, Room 118, Stanford, CA 94305 USA; 4 Department of Earth, Environmental, and Planetary Sciences, Brown University, Providence, RI 02912 USA; 5 Institut des Sciences Exactes et Appliquées, Université de la Nouvelle-Calédonie, BP R4, 98850 Nouméa, Nouvelle-Calédonie; 6 1150 4th St SW Apt 1005, Washington, DC, 20024 USA; 7 Royal Botanic Gardens Victoria, Birdwood Avenue, Melbourne, VIC 3004 Australia; 8 Service Géologique de la Nouvelle-Calédonie, 1 ter rue Unger, BP M2, 98849 Nouméa Cédex, Nouvelle-Calédonie; 9 Missouri Botanical Garden, 4344 Shaw Blvd., St. Louis, MO 63110 USA; 10 Oak Spring Garden Foundation, 1776 Loughborough Lane, Upperville, VA 20184 USA; 11 Histoire Naturelle de L’Homme Préhistorique, Muséum National d’Histoire Naturelle, CNRS, Sorbonne Université, UPDV, 1 rue René Panhard, 75013 Paris, France; 12 Institut Méditerranéen de Biodiversité et d’Ecologie Marine et Continentale, (UMR CNRS 7263/IRD 237), Centre IRD de Nouméa, BP A5, 98848 Nouméa Cedex, Nouvelle-Calédonie

**Keywords:** Evolution, Palaeontology

## Abstract

New Caledonia was, until recently, considered an old continental island harbouring a rich biota with outstanding Gondwanan relicts. However, deep marine sedimentation and tectonic evidence suggest complete submergence of the island during the latest Cretaceous to the Paleocene. Molecular phylogenies provide evidence for some deeply-diverging clades that may predate the Eocene and abundant post-Oligocene colonisation events. Extinction and colonization biases, as well as survival of some groups in refuges on neighbouring paleo-islands, may have obscured biogeographic trends over long time scales. Fossil data are therefore crucial for understanding the history of the New Caledonian biota, but occurrences are sparse and have received only limited attention. Here we describe five exceptional fossil assemblages that provide important new insights into New Caledonia’s terrestrial paleobiota from three key time intervals: prior to the submersion of the island, following re-emergence, and prior to Pleistocene climatic shifts. These will be of major importance for elucidating changes in New Caledonia’s floristic composition over time.

## Introduction

Integrating biological and geological knowledge is crucial for understanding biotic evolution^1^ and recent reviews^2–6^ have emphasized the need for additional paleontological data to enable more powerful tests of evolutionary and biogeographic hypotheses. Despite New Caledonia’s importance as a global biodiversity hotspot, such an integrative synthesis has not yet been fully developed for this Pacific archipelago, mainly because of a lack of detailed paleontological studies. New Caledonia has long fascinated biologists because of its isolated flora and fauna, but a deep understanding of the evolution and assembly of this biota has remained controversial because biological and geological data appeared to be in conflict regarding its age. The New Caledonian biota contains representatives of some deep-branching lineages, including *Amborella*, the sister group of all other Angiosperms. However, recent geophysical, tectonic, and sedimentological studies^7,8^ suggest that the extant island is not nearly as old as many of these lineages and that the ancestors of its modern biota must have migrated more recently from other regions^2,4–6^. These studies suggest a complex history, with the complete submergence of what is currently New Caledonia during the Late Cretaceous and Early Paleogene, possible sporadic re-emergence(s) during pre- and syn-obduction tectonic events between ~ 50 MY (millions of years) and ~ 34 MY^9^, and finally, full emergence following obduction at some time between 34 and 25 MY^8,10^. Following this period of uplift, an ultramafic regolith developed over peridotites throughout the island, and owing to its specific geochemical features, dramatically controlled terrestrial colonization. Although evidence for erosion of an older regolith was described from early Miocene sediments^10,11^, the ancient character of regolith development has generally been overlooked.

The geology of New Caledonia has been considered unfavourable for fossilisation. Sediments from the Permian to the Quaternary are present on the main island, but many are metamorphosed and large areas are covered by mantle peridotites resulting from obduction. Terrestrial fossil assemblages are therefore rare and very few have been documented. Among animals, some Holocene vertebrates and land snails are known^12–14^. Plants are better represented, but mainly by pollen and wood from scattered Permian to Holocene deposits^15–22^. Macrofloral remains are likewise rare, with a few Late Cretaceous angiosperms and leaves of the gymnosperm *Podozamites* mentioned in old literature^21^, together with a probable leaf of Ginkgoales and leaves of *Taeniopteris* from the Triassic^18^. A variety of leaves, stems, and unidentified roots have also been reported form Miocene ferricretes derived from weathered peridotites^23^, but more precise identifications were not possible. Subfossil copal resins have also been found, but without identifiable inclusions (RG pers. obs.). This record is too sparse to provide a clear understanding of the evolution and assembly of terrestrial paleobiotas before and after New Caledonia’s submersion and subsequent re-emergence^24^.

**Figure 1:**
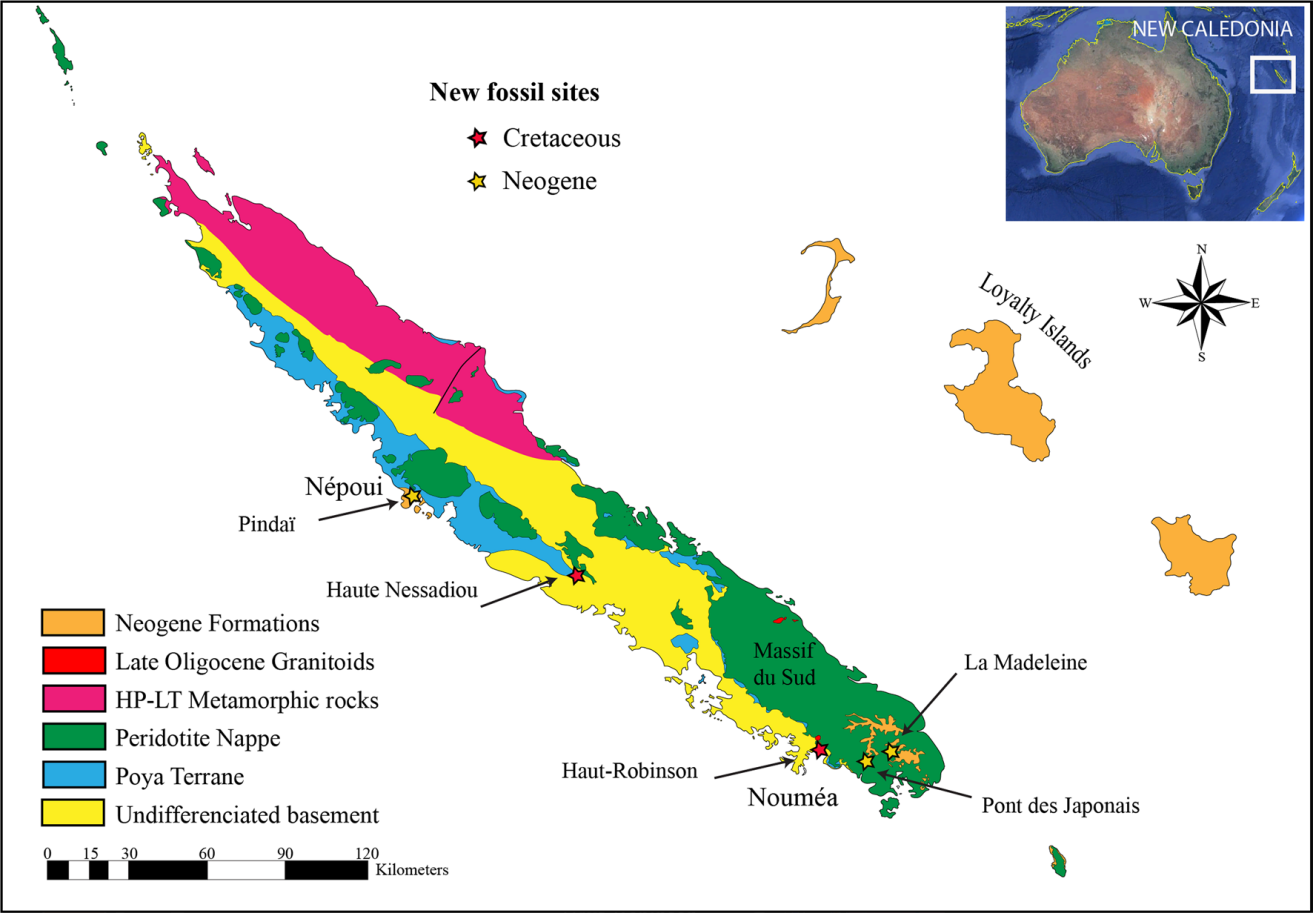
Simplified geological map of New Caledonia with locations of new fossil outcrops. Copyright P. Maurizot.

To address the lack of a detailed fossil terrestrial record, our research focused on potentially fossiliferous terrestrial sedimentary outcrops, particularly those likely to preserve plants and insects. New fossil assemblages were discovered across New Caledonia, from the Upper Cretaceous ‘Formation à charbon’ of Moindou (northern part of South Province), the Upper Cretaceous of Haut-Robinson (Mont-Dore municipality), the lower Miocene of Nepoui (southwest coast of North Province), and the middle(?) Miocene of the Fluvio-Lacustrine Formation (Madeleine falls and ‘Pont des Japonais’ outcrops, South Province) (Fig. 1 and Supplementary Figs. 1,4,7,9). These discoveries dramatically expand the known diversity of plants and insects from the Cretaceous through the Neogene, provide important insights into past ecosystems on New Caledonia, and show that the island does indeed possess a diverse and widespread paleontological record that helps to illuminate key moments in its geological, evolutionary, and ecological history. These floras and faunas serve as a crucial first step towards a more integrated understanding of the complicated history of the New Caledonian biota.

## Results

The geologic context of the newly discovered fossil-bearing outcrops found across New Caledonia (Fig. 1, and Supplementary Figs. 1,4,6,8), their basic features, and their preserved biota are discussed below. As a matter of reference, the New Caledonia archipelago comprises a main island (Grande Terre), its offshore extensions (Isle of Pines and Belep Islands), and a girdle of smaller and much younger islands (Loyalty Islands) that extends parallel to and east of the main island. The Grande Terre is the emerged northernmost part of the ‘microcontinent’ Zealandia^25^.

### Late Cretaceous of Haute-Nessadiou (H-Ness) and Haut-Robinson (H-Rob)

**Figure 2:**
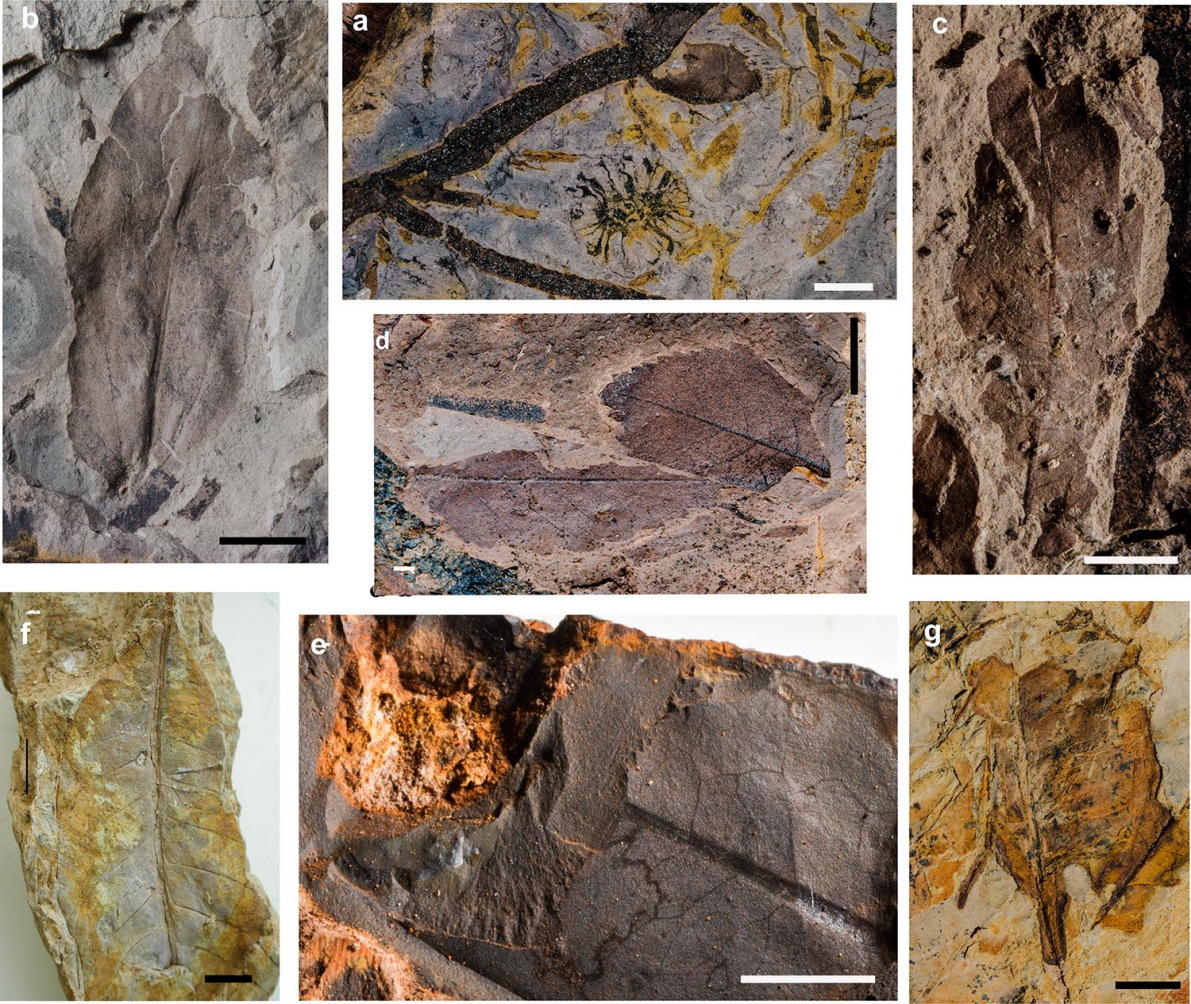
Fossil plants and insect activities. (**a**) cone of taxodiaceous Cupressaceae; (**b–d**) leaves; (**c**) insect activities on a leaf (Late Cretaceous, Haute Nessadiou); (**e**) insect activities on leaves (Late Cretaceous, Haut-Robinson); (**f, g**) insect activities on leaves (Nepoui, Early Miocene); Copyright R. Garrouste. Scale bars = 1 cm.

The Upper Cretaceous sedimentary cover of Grande Terre consists of a passive margin megasequence, with coarse detrital terrestrial to marine peri-continental sediments at its base and fine-grained marine transgressive deposits towards the top. We located several fossiliferous outcrops within this sequence in the Haute-Nessadiou area between village of La Foa, Boghen pass, and the village of Moindou. This area contains a NW–SE striking strip of Upper Cretaceous mudstones and thin sandstones, 4 km wide and 16 km long (‘Formation à charbon’, Supplementary Fig. 1) that reflect distal fluvial deposition. Marine bivalves and gastropods indicate deposition in estuarine marine environments while coal seams and other mudstone units contain in situ root horizons, indicating terrestrial deposition and development of soils. Marine invertebrate biostratigraphy (Supplementary Information) supports a Turonian to late Santonian age (~ 90–85 MY). Plant fossils occur in millimeter-thick argillite beds in several places and are dominated by conifers, including a woody ovuliferous cone scale with a distinct free tip consistent with the family Araucariaceae, and numerous cone scales in a complex expanded distally to form a distinct umbo as in extant genera such as *Sequoia* and *Sequoiadendron*, indicative of taxodiaceous Cupressaceae (Fig. 2a). Conifer foliage includes small needle-leaved taxa as well as broader leaved forms with a single midvein. One poorly preserved fern specimen and a possible cycad sporophyll with attached seeds were also recovered. At least five taxa of flowering plants (angiosperms) have been found in these deposits, including forms with both entire and toothed margins from moderately sized to large leaves (Fig. 2b-d). Precise systematic placement of these plant fossils is difficult because the cuticles and fine venation are not preserved, and because of the fragmentary nature of the material and thermal alteration of the sediments. No fossil insects were recovered from these sediments, but a number of the angiosperm leaves show marginal feeding traces, galling, and mining made by insects (Fig. 2c,e). Typically, arthropod remains are rare in soft mudstone and non-consolidated sandstone sediments^26^.

The Haut-Robinson plant outcrop is contained within the same general sequence as that at Haute-Nessadiou. Here the base of the Upper Cretaceous succession is interbedded with volcanic agglomerates, flows, and sills 10 to 50 m thick (Supplementary Fig. 1) derived from the trachyte and rhyolite flows that directly overlie the Jurassic basement^27^. Fluvial sandstones and siltstones in this outcrop contain abundant small woody stems and other fragmentary evidence of vegetation. Leaf fossils are rare and are confined to a few horizons. The material includes at least three taxa of ferns assigned to *Microphyllopteri*s, *Cladophlebis*, and *Sphenopteris* (Supplementary Fig. 3). Similar fossil foliage is generally placed in Gleicheniaceae, Osmundaceae, and Dicksoniaceae, respectively. Small needle-leaved conifers, including foliage from *Elatocladus* and *Pagiophyllum*, are also present. A possible cone scale (*Araucarites*) also suggests the presence of araucariaceous conifers. Angiosperm leaves are small and rare, but at least two taxa are present. The first has craspedodromous-like venation with the main secondaries terminating in a major tooth at the leaf margin (Supplementary Fig. 3 g). Similar types of leaves occur in the fossil assemblage from the Cretaceous Winton Formation of Australia^28^. The second taxon has larger leaves and more steeply/shallowly angled venation. The flora of the Haut-Robinson outcrop is distinct from that of the Haute-Nessadiou Formation, probably due to the more proximal mode of deposition. No fossil insects have been found at the Haut-Robinson plant outcrop, but the fossil leaves show numerous mines and galls.

### Early Miocene of Nepoui Group and Middle(?) Miocene of the Fluvio-Lacustrine formation

In the absence of unconformably overlying younger sediments, obduction in New Caledonia is not precisely dated; however, there is a consensus that the post-obduction period started in the early Oligocene^7,8^. The oldest known aerial exposures of weathered ultramafic rocks (ferricretes) have been dated by paleomagnetism from the Late Oligocene^29^. The earliest terrestrial deposits containing fossils (dating to the Early Miocene) are therefore very important for understanding the post-obduction recolonisation process and elucidating the origin of key extant lineages. The Early Miocene deposits of the Nepoui Formation, as well as Middle(?) Miocene deposits of the Fluvio-Lacustrine Formation, both contain diverse plant remains, although with only a few insect traces of activity or other terrestrial animals (see below). Further field collections are needed to fill this gap.

### Nepoui group

The Nepoui Group is centered around the Pindai Peninsula on the west coast of Grande Terre. It comprises two formations^30^: the lowermost part of the lower formation is a ca. 100 m thick sequence of reefal and lagoonal limestones, reflecting the earliest post-obduction sedimentation (Supplementary Fig. 4). This is overlain unconformably by coarse conglomerates, ca. 100 m thick that grade upwards into a finer-grained ‘intermediate unit’ with both marine and terrestrial sediments. The intermediate unit is approximately 15 m thick and is made up of alternating bioclastic and lithoclastic sands, conglomerate lenses, and calcareous mudstone. Numerous imprints and molds of fossil leaves, as well as a few insects, occur in these beds. Silicified and ferruginous fossil wood fragments and silicified infructescences corresponding to extant *Gymnostoma* (Casuarinaceae; see below) are also common in the conglomerate^31^ (Supplementary Figs. 5j-k). These beds grade upward into the upper Nepoui Formation, which consists of about 25 m of bioclastic limestones that are rich in coral, algae, and echinoid fragments (Supplementary Information). The ages of both the lower and upper limestones are Early Miocene, based on planktonic and benthic foraminifera. Specifically, the Upper Nepoui Formation has been dated to the Aquitanian–Burdigalian (21.4–17 MY), constraining the age of the plant fossils that occur immediately below it. We discovered a fossiliferous plant layer, approximately 10 cm thick within the intermediate unit (Figs. 2f,g, Supplementary Fig. 5), that consisted of dense mats of leaves, suggesting an allochthonous accumulation. Preliminary analysis reveals a diverse floral assemblage consisting of more than 40 distinct leaf morphotypes, including around 40 dicot angiosperm leaf morphotypes, one scale-leaved conifer, and a fern, from a very small total collected exposure (ca. two square meters of rock) (Supplementary Information). The variety of forms in a limited exposure, as well as the representation of most morphotypes by only a single specimen, suggests that the Early Miocene source vegetation in this part of New Caledonia was highly diverse (Supplementary Information). The fossil leaves are difficult to assign to extant angiosperm genera or families because of their relatively poor preservation, but two types of woody infructescences are assigned to *Gymnostoma* (Casuarinaceae). The larger type, with large prominent bracteoles and large subtending bracts, is similar to infructescences assigned to extant *Gymnostoma* from the Eocene of Australia and Argentina^32,33^. The smaller type is more poorly preserved, but is also consistent with *Gymnostoma* in its prominent bracteoles that are more widely separated than in *Casuarina* or *Allocasuarina*. Traces of insect activity occur frequently on the angiosperm leaves (margin feedings, galls; Fig. 2g). Compared to plants living in the extant dry, sclerophyll forest of the Pindai Peninsula today, the leaves of Miocene angiosperms were generally larger, suggesting greater precipitation (Supplementary Information). Together with the presence of *Gymnostoma*, which is abundant on New Caledonia but not in the relatively dry Pindai Peninsula, paleobotanical data are congruent with previous suggestions of higher rainfall in New Caledonia during the Early Miocene^34^.

### The Fluvio-Lacustrine formation (Fig. 1) (Supplementary Information)

**Figure 3:**
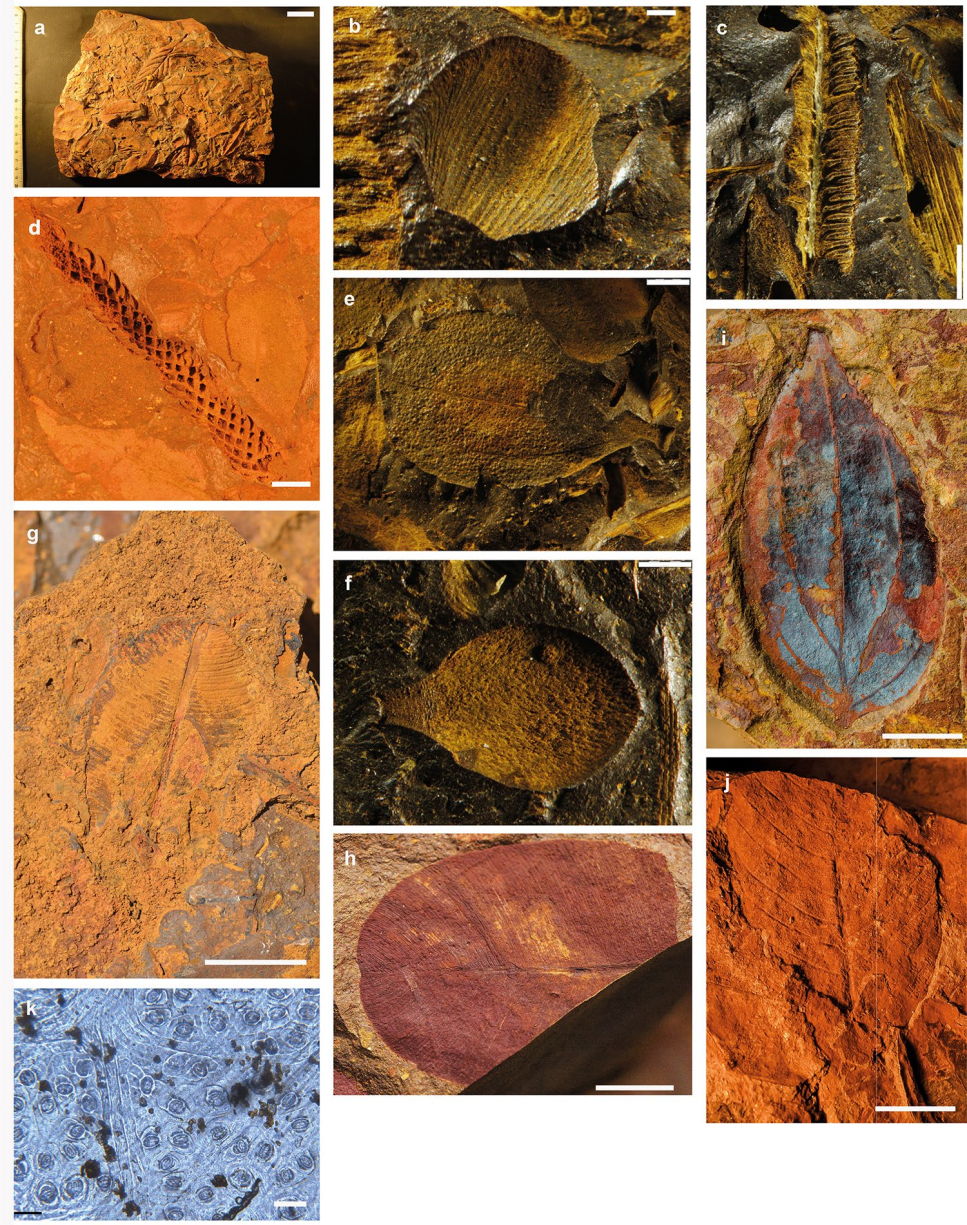
Middle(?) Miocene plants. (**a–f**) La Madeleine outcrop; (**g–j**) Pont des Japonais outcrop. (**a**) Mad_1 from Madeleine showing the very dense fossils assemblage; (**b**) inflorescence bract of *Styphelia* sp. (Ericaceae ex-Epacridaceae), Mad_1_A_Fe142; (**c**) fern pinnule, Mad_1_Fe49; (**d**) axis with 48 leaves of cf. *Dacrydium* sp. (Podocarpaceae), Mad_17_A_Axe1; (**e**) leaf of *Melaleuca* sp. (Myrtaceae), Mad_1_A_Fe171; (**f**) probable petal of dicotyledon, Mad_1_A_Peta115; (**g**) leaf of *Solmsia* sp. (Thymelaeaceae), Mad_22_A_Fe1; (**h**) leaves of *Calophyllum* cf. *caledonicum* (Calophyllaceae), Pjap_21_A; (**i**) leaf very similar to that of an extant species of *Cryptocarya* (Lauraceae), Pjap_46_A_Fe1; (**j**) leaf of *Alphitonia* cf. *caledonica* (Rhamnaceae), Pjap_55_A_Fe2; (**k**) imprint of Mad_6_Fe1 showing epidermis and stomata (same type of leaf as specimen figured in 3i). Copyright J. Munzinger (**a, h, i**); Copyright Thibault Chaillon (**b-f**); Copyright R. Garrouste (**g**); Copyright Wyndy Foy (**j,k**). Scale bars (**b**) & (**f**) = 1 mm; (**e**) & (**c**) = 2 mm; (**d**) = 5 mm; (**k**) = 50 µm.

**Figure 4:**
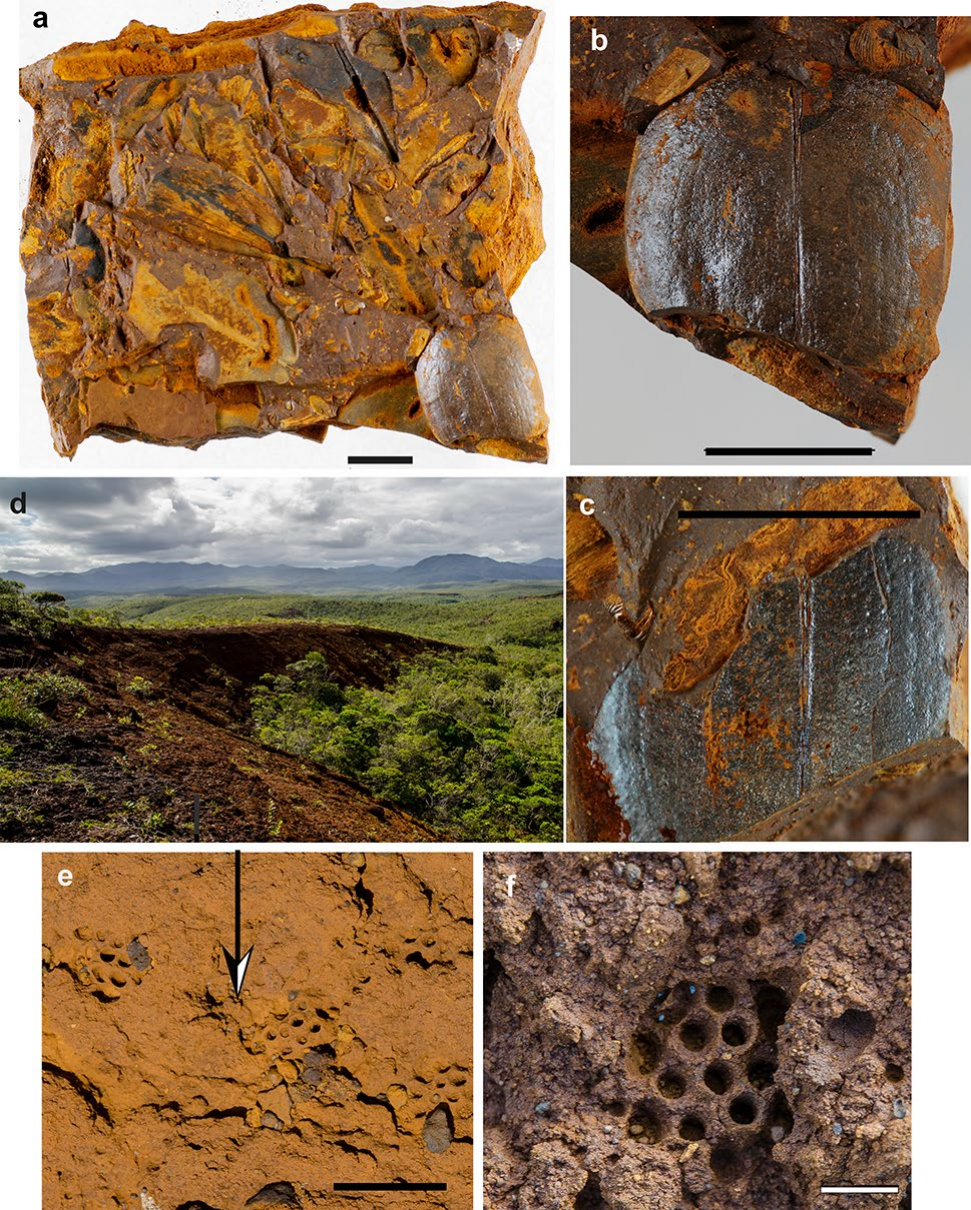
Middle(?) Miocene ferricrust with plant and insect remains. (**a–c**) ‘La Madeleine’ outcrop; (d-g) ‘Pont des Japonais’ outcrop. (**a**) slab with non-oriented plants. NC. MAD 01 A; (**b**) detail of a pair of elytra of a beetle (Coleoptera: Scarabaeoidea). NC. MAD 01 A; (**c**) details of counterpart (NC. MAD 01 B); (**d**) ‘Rivière des Pirogues’ sedimentary basin landscape and outcrop, ‘Pont des Japonais’ area with ichnofossils (*Rosellichnus* sp.); (**e**) details of clustered ichnofossil *Rosellichnus* sp. on subsurface of the latericrust; (**f**) ichnofossil *Rosellichnus* sp. attributed to eusocial Hymenoptera NC-PJ-O2. Copyright R. Garrouste. Scale bars (**a–d,f**) = 1 cm; (**e**) = 4 cm.

This formation comprises the sedimentary infill of depressions mainly located in the Massif du Sud in southern Grande Terre, which includes the Yaté Basin, Plaine des Lacs, Rivière des Pirogues, and Creek Pernod^35^. This unit, 70 to 80 m thick, formed from the erosion of weathering profiles that developed over peridotites or gabbro cumulates^36,37^. It also displays evidence of hydromorph (palustrine) pedogenesis with horizons of ferric crust and includes plant roots encrusted with iron oxides, as well as localized layers that are rich in well-preserved fossil plant remains (Figs. 3 and 4). Over most of its area, it is capped by ferruginous cuirasses (Plateau de Gertrude, La Madeleine waterfall, etc.) that probably formed in association with an ancient water table. Fossiliferous layers are distributed throughout the whole series, including in some cuirasses (La Madeleine waterfall). The general pattern of the fossiliferous sites is broadly similar to fossiliferous travertine formations, and a few cases of iron travertine deposits are known in Spain^38,39^ and in Greece, also on ultramafic formations with ferrihydrite encrusted leaves^40^.

The precise age of these deposits is difficult to determine. Attempts at paleomagnetic dating of internal duricrusts and lateritic ferricrete on top of the sequence yielded contradictory results ranging from latest Oligocene (25 MY) to Miocene (15 MY). Folcher et al*.*^41^ proposed several age hypotheses for the Fluvio-Lacustrine Formation, spanning the latest Oligocene to Holocene (ca. 120,000 years ago). They favour a possible early to middle Miocene age by correlation with tectonic events recorded in Nepoui sediments (coarse conglomerate and erosion sequence positions). We were unsuccessful in our attempt to use electron spin resonance (ESR) to date quartz grains at the two major fossil localities (La Madeleine and the Pont des Japonais), where quartz grains are exceedingly rare.

At the ‘La Madeleine’ locality (Mad), poorly preserved casts of leaves and wood fragments occur at the surface of a ferruginous cuirass along the La Madeleine River close to its falls on the Plaine des Lacs. More productive, however, is a small (~ 10 m long) but rich fossil-bearing layer of ferricrust that occurs in a small hill near the Madeleine waterfall (Supplementary Fig. 7). This matrix preserves plant remains in three dimensions and with cellular detail, including leaves, flowers, seeds, and wood (Supplementary Fig. 8). These fossils are preserved as dense mats of plant material that accumulated in great quantity in small sinks, without particular orientation, suggesting deposition in the absence of a current. They consist mainly of eudicot leaves (Figs. 3 and 4) with additional remains of flower petals, inflorescence bracts, and seeds, as well as remains of ferns and gymnosperm branches with attached leaves. Although original organic material is not present in these fossils, some show exceptional preservation of epidermal and anatomical details, including stomata, which should help to improve future identification. Preliminary results indicate the presence of at least 30 plant morphotypes, including likely representatives of the angiosperm families Ericaceae (ex Epacridaceae), Malvaceae-Sterculioideae, Thymelaeaceae, and Myrtaceae, but further investigation of more material is needed. Some fossil representatives are nearly identical to the leaves of extant taxa of Thymelaeaceae (*Solmsia*) and Ericaceae (*Styphelia*). Leaf physiognomy and the taxonomic composition of the fossil flora at the La Madeleine site are strongly consistent with the present-day local maquis vegetation, suggesting that similar plant communities may extend as far back as the late Miocene^42^. The La Madeleine site also preserves a three-dimension cast of a pair of beetle elytra, which were found in association with a dense leaf mat (Fig. 4a-c). These remains are attributed to an extinct species of scarabaeid beetle, likely related to an extant New Caledonian species found in the rainforest of the same area (Rivière Bleue) (Supplementary Information).

The ‘Pont des Japonais’ (PJap) outcrop includes several fossiliferous layers along several hundred meters of exposure on a road to the Pont des Japonais. As at ‘Mad’, fossils are preserved in three dimensions in iron oxide and include leaves, seeds, and wood fragments (Fig. 3 and 4). The density of fossils is lower than at ‘Mad’ and leaves are not typically found as dense mats, but the ‘PJap’ assemblages nonetheless contain a high diversity of leaf morphotypes. The composition of the flora is also different, and includes gymnosperms, Calophyllaceae, Ericaceae (ex Epacridaceae), Lauraceae, Malvaceae-Sterculioideae, and Rhamnaceae. The leaf morphology and taxonomic affinities of the ‘PJap’ fossils, especially the abundant leaf fossils that resemble those of extant New Caledonian Lauraceae, are consistent with a rainforest community similar to that of the current vegetation in the ‘Rivière Bleue’ reserve in the southern Grande Terre^43^. In general, the ‘PJap’ fossil leaves are also larger than those at ‘Mad’, further consistent with a rainforest rather than a maquis community. The ‘PJap’ assemblages also contain some evidence of insects; feeding marks are present on a few leaves (margin and galls), one layer contains numerous wasp nests (Fig. 4d-f), and another ca. 4 m thick layer contains numerous bee nests built with consolidated mud and very small fragments of iron oxide. We attribute these nests, comprising clusters of cells that open to flat surfaces, to the ichnogenus *Rosellichnus* Genise and Bown, 1996^44^. The contemporaneity of such ichnofossils with the rocks that contain them is difficult to establish^45^, but the absence of any organic remains in them suggests they are fossils.

### Comparison of fossil assemblages

The new discoveries suggest that the Late Cretaceous floras from New Caledonia are generally similar to those from contemporaneous eastern Gondwanan communities but contribute important new data, because such floras are rare. In Australia, the Winton Formation from central Queensland, which spans the Latest Albian or Cenomanian to early Turonian, is one of the few well-known macrofloras in the region^28,46^. Other Late Cretaceous floras include that from the Waare Formation in the Otway Basin in Central Australia^47^. New Zealand Late Cretaceous is better represented with floras of the Albian to Cenomanian (Clarence Series)^48^, Cenomanian to Turonian (Tupuangi Formation)^49,50^, Campanian (Taratu Formation)^51^ and Latest Cretaceous (Pakawau Group)^52^. Comparison of the New Caledonia assemblages to these floras confirms the widespread occurrence of taxodiaceous Cupressaceae, which dominate in distal deltaic settings (such as the Pitt Island sequences of the Tupuangi Formation), suggesting their importance in coastal environments. This prominence of conifers during the mid- to Late Cretaceous is consistent across the Gondwanan margin to the Antarctic Peninsula, where they occur in distal facies in the late Albian Alexander Island flora^53^.

The rise of flowering plants during the mid-Cretaceous is not well documented in eastern Gondwanan macrofloras, but angiosperms are important in Late Cretaceous floras, including those of New Caledonia. The first Australian angiosperms appeared toward the end of the Early Cretaceous^54^ and are widespread in Late Cretaceous assemblages. Late Cretaceous angiosperms from New Caledonia are similar to those of the Winton Formation of Queensland in general leaf physiognomy and especially in the predominance of small-toothed morphotypes. This contrasts with approximately coeval floras from New Zealand such as from the Clarence Series, which contains larger and entire-margined leaves, although it is unclear if this difference reflects contemporaneous spatial heterogeneity in climate and community composition or floras of slightly different ages. The presence of angiosperms in these communities is also associated with pronounced evidence of insect herbivory in the form of leaf damage, although no insect body fossils have been recovered from New Caledonia.

The Early Miocene Nepoui flora is especially important as the first known post-obduction terrestrial paleobiota. Our results suggest this deposit preserves a highly diverse angiosperm community growing in a more humid climate than is found on the western coast of Grande Terre today, which is consistent with reconstructed paleoclimates^34^. The rich and diverse flora, together with the numerous traces of interactions with arthropods (Fig. 2f,g), are indicative of a complex paleobiota, suggesting extensive re-colonisation of the island potentially only ca. four million years following its final re-emergence. This Miocene fossil material also directly establishes the presence of some present-day groups in New Caledonia (e.g., *Gymnostoma*). The younger outcrops of the Fluvio-Lacustrine Formation document a rich, diverse, and complex tropical paleobiota. The diversity of leaf morphologies in the Fluvio-Lacustrine Formation, from those typical of wet forests at the Pont des Japonais to those at La Madeleine that are nearly identical to modern species living in ultramafic ‘maquis’ communities, demonstrates the existence of a diverse set of communities in southern Grande Terre during the Neogene, at least well before the arrival of humans. Such a mosaic landscape is still present today, in part maintained by wildfires before the last 50,000 yr^55,56^ or, more recently, by fires of human origin.

Based on new fossil evidence, it is clear that the modern vegetation of New Caledonia differs fundamentally in composition from its Late Cretaceous plant communities, as well as those from eastern Gondwana more generally, and not only in a general shift away from conifer dominated lowland settings to diverse angiosperm forests. New Caledonia cannot be considered as a kind of Mesozoic Gondwanan ‘refuge’ and its flora thus appears to have undergone similar kinds of changes as the broader biogeographic region in which it is situated, at least in terms of a basic taxonomic turnover towards angiosperm-dominated communities over long geological periods^57^. Present fossil evidence does not rule out the possibility that some of the characteristic deep-branched lineages endemic to New Caledonia (e.g., *Amborella*) are relicts of earlier ecosystems (viz. those of Zealandia) that potentially survived obduction on neighbouring now-drowned islands, as suggested by geological studies^58,59^ and biogeographic analyses based on fossil and present-day taxa^2,3,60–62^. Nevertheless, assembly of the modern New Caledonian biota from the Oligocene to early Miocene is most consistent with the ages inferred by molecular dating for many extant clades^6^ and the idea that much of the current biota was assembled via dispersal from elsewhere. Our fossil evidence suggests that highly diverse angiosperm communities, very similar to those found on New Caledonia today, were established on the island by the early Miocene. It also shows that these communities evolved during the Miocene as the plant assemblage of Nepoui is markedly different from those of Pont des Japonais and La Madeleine. These fossil assemblages provide an important new minimum age for the assembly of the modern New Caledonian flora. On the basis of the current material and further study of these sites, as well as investigation of potential new sites, it will be possible to develop a detailed inventory of New Caledonia’s Miocene flora and entomofauna, which will aid the molecular dating of modern clades and provide insight into the taxa that disappeared during regional aridification over the last 20 MY.

## Methods

### Materials

All material collected is the property of the Government of New Caledonia, Service Géologique de la Nouvelle-Calédonie (SGNC), and is temporarily deposited in the MNHN Paris, the Geological Sciences Department at Stanford University, and the Royal Botanic Gardens Victoria, Australia.

### Imaging

The observations were made under a binocular microscope and photographs were taken with a digital SLR (NIKON D800 with a 60 mm f2.8 Nikkor lens) camera with a controlled oblique light. SEM microscope images were produced with BSE mode and EDS X-ray analysis (service des collections, MNHN and Yale University).

## Supplementary Information

Supplementary Information 1.

Supplementary Information 2.

## Data Availability

All relevant data are available from the authors.
