# Supplementary material for: New fossil discoveries illustrate the diversity of past terrestrial ecosystems in New Caledonia
Source: Sci Rep. 2021 Sep 15;11:18388. doi: 10.1038/s41598-021-97938-5 (PMC8443626; doi:10.1038/s41598-021-97938-5)
Supplement: Supplementary file 1 — Supplementary Information 1. [file 41598_2021_97938_MOESM1_ESM.pdf]

Supplementary information

New fossil discoveries illustrate the diversity of past terrestrial ecosystems in New Caledonia

Romain Garrouste[1], Jérôme Munzinger[2], Andrew Leslie[3], Jessica Fisher[4,deceased], Nicolas Folcher[5], Emma Locatelli[6], Wyndy Foy[2], Thibault Chaillon[2], David J. Cantrill[7], Pierre Maurizot[8], Dominique Cluzel[5], Porter P. Lowry II[1,9], Peter Crane[10], Jean-Jacques Bahain[11], Pierre Voinchet[11], Hervé Jourdan[12], Philippe Grandcolas[1] & André Nel[1,*]

Material and methods.

Several field trips have been conducted since the first discoveries of Miocene fossiliferous deposits in April 2011 by RG, AN, PM, DC. A representative sample of fossil material was collected from each deposit for study in the laboratory, and additional samples were also made for studies of fossil animals (insects, interaction traces) and taphonomy. The position of the fossil layers was identified in stratigraphic geo-localized logs. Putative modern analogue environments (e.g., lakes, sinkholes) were also examined to help understand taphonomic processes, including the exceptional fossilization by iron oxides of Miocene material from the Madeleine (Fluvio-Lacustrine Formation).

Various dating methods have been used, but none has given reliable results with appropriate confidence intervals yet (discussed in the text). In order to precisely date sediment deposition, three samples were tested for Electron Spin Resonance (ESR) dating. Two of them ('Pont des Japonais' and 'la Madeleine' sites) corresponded to very fine indurated and iron-rich sediments (silts and clays), whereas the third one (also from 'la Madeleine') contained coarser grains in sandy silt sediments. However, none provided enough quartz grains to allow ESR dating. Sediment samples for pollen analysis, collected from the various layer studied, or adjacent layers, have also proved unproductive.

Extended geological information about the new fossil outcrops

Late Cretaceous period (Supplementary Figs. 1-3)

From the Late Cretaceous to the Paleocene, several marginal basins separating thinned continental crust fragments opened on the eastern Gondwana margin above a west dipping subduction zone which propagated toward the Pacific via trench rollback process[1-3]. The easternmost known continental fragment is the Norfolk ridge which bears both New Caledonia and New Zealand, sharing the same pre-Paleogene geological history. As a consequence, the Late Cretaceous sediments were deposited unconformably over the previous amalgamated basement (Upper Permian to Lower Cretaceous greywackes) during or after this period of rifting and expansion. The Upper Cretaceous sedimentary cover of the Grande Terre of New Caledonia consists of a passive margin megasequence, with coarse detrital terrestrial to marine peri-continental sediments at the base (syn-rift deposits, currently referred to as 'Formation à charbon') and fine grained marine transgressive deposits towards the top (post-rift deposits).

Sediments of this period are well represented in several areas on the SW flank of the Central Range, near La Foa and around Nouméa (Supplementary Fig. 1).

In the area between the village of La Foa and the Boghen pass a strip of Late Cretaceous, striking NW-SE, 4 km wide and 16 km long, is exposed. The basal conglomerates are well developed on the NE flank of the structure and passes gradually to the SW to finer grained sandstones. This asymmetrical arrangement evokes infilling of a half-graben with a steep border fault to the NE.

In the area of Nouméa (Robinson), there is no basal conglomerate and the base of the Late Cretaceous succession is interbedded with volcanic rocks represented by agglomerates,

flows and sills, 10 to 50 m thick. Trachyte and rhyolite flows directly overlie the Jurassic basement[4]. The compositions are basaltic, andesitic, and trachytic (ignimbrite). Mafic and felsic compositions are most frequent at the expense of intermediate one. All lateral equivalent as pyroclastic (agglomerates, breccia, crystal and lithic tuffs, and ash fall) or epiclastic rocks (volcaniclastic sandstones, conglomerate) are present. The bimodal composition of the lavas is typical from syn-rift volcanism.

In both areas, the Upper Cretaceous succession is structured into large SW verging overturned to thrust folds, the coal seams playing the role of 'decollement' levels, hampering stratigraphic detailed analysis and excluding accurate thickness estimate. Nevertheless, it is possible to establish a common and broad stratigraphic succession.

The coarse conglomerate contains predominantly ungraded angular clasts of the basement. The conglomerate matrix and the cortex of some of the boulders are ferruginous and oxidized, indicating subaerial evolution. They change upward into roughly bedded, but finer and better graded conglomerate containing well rounded pebbles of the same components. The unit grades into quartz rich micaceous and arkosic sandstone. Elementary sequences of several meters thick are formed by a typical fining upward floodplain succession: micro-conglomerate, sandstone, mudstone, coaly mudstone, and coal. Sulphidized wood fragments, up to 50 cm, are common. The sandstones exhibit trough cross-laminations with erosive tops. Clay-drape couplets, symmetrical ripples, herringbone cross-beds, tabular or trough cross bedding are common features indicating a coastal and tidal influence. The plant fossils have been found in these millimeter-thick argillite beds. Marine bivalves bearing sandstones are intercalated occasionally in these sequences. These deposits characterize a coastal environment from continental to shallow marine, including fluvial and deltaic sediments.

These terrigenous sediments fine upward and grade into a succession of black siltstones and argillites with abundant organic matter, sulphides and fossilliferous nodules. They are

referred to as the 'Mamelons Rouges beds'[4]. In turn, the black argillites become increasingly siliceous and grade upwards into decimetre-thick undulating and nodular black chert beds. These fine-grained sediments were deposited in a hemipelagic to pelagic context resulting from the post-rift thermal subsidence[5] that drowned the structures, with a subsequent considerable reduction in terrigenous input and anoxia.

The Late Cretaceous formations of New Caledonia have their equivalents in New Zealand: the 'Coal measures' for the 'Formation à charbon' and the 'Whangai formation' for the 'black cherts'[6].

A late Early Cretaceous (Albian) zircon population is appearing in several basement terranes of New Caledonia (Maurizot, pers. obs.). This last age is therefore considered as the oldest possible age for the Late Cretaceous succession. Although the macrofauna consisting of ammonites, bivalves, and gastropods is relatively abundant, especially in the 'Mamelons Rouges beds' nodules, it is quite difficult to date precisely these levels due to the high endemism of biota. For this period, several ammonite informal zones have been recognized in New Zealand that could be extended to New Caledonia; however, these zones are too poorly known to be useful for correlations with the international time scale[7]. The inoceramid bivalves are of much utility for their wide distribution and high evolutionary rates. Commonly reported species for the coal bearing mid unit are *Inoceramus pacificus* Woods, *I. opetius* Wellman, *I. bicorrugatus* Marwick and *I. australis* Woods which are distributed from the middle Turonian to the Santonian. The 'Mamelons Rouges beds' macrofauna is assigned to the Campanian interval whilst the black cherts are assigned to the late Campanian – Maastrichtian[8]. Volcanic rock from the syn-rift volcanism has been dated in the Nouméa region by U/Pb method on zircon[9-10]. The ages range from 190 to 76 MY. 14 ages are in the interval 76 to 97 MY (Cenomanian to middle Campanian) with an average of 84.8 MY. Older ages are likely measured on zircon grains inherited from the basement. The 'Formation à charbon', in which

plant fossils have been discovered, is thus assigned to the late Turonian to early Campanian interval.

Some indications on the paleogeographic situation of New Caledonia during the deposition of the 'Formation à charbon' may be inferred from provenance of the terrigenous input[11-12] especially zircons. The younger zircons are contemporaneous with the Late Cretaceous volcanic activity. A mid-Cretaceous population (110–95 MY) is yet not explained, rocks of this age being rare in New Caledonia[13]. The ages of the older detrital zircons in the 'Formation à charbon' reflect the ages of the zircons present in the underlying basement including Early Paleozoic and Precambrian ages. Therefore a local recycled provenance for the Precambrian detrital zircons of the Late Cretaceous syn-rift sediments can be contemplated instead of a direct Australian provenance[14]. New Caledonia was likely isolated from Australia by the beginning of Late Cretaceous, feature consistent with the faunal endemism. Although the deposition of the 'Mamelons Rouges beds' and the subsequent black cherts corresponds to a period of drowning, a complete submersion of the northern tip of the Norfolk Ridge at this time is not likely since plant remains are common in this sediments.

Miocene period (Supplementary Figs. 4-9)

On the Grande-Terre, the post-obduction period is characterized, by extensional fault movements[15] that led to the uplift of the Norfolk Ridge and collapse of its margins. Several formations are defined: either weathering profiles or sediments. Thick regolith developed on the emerging peridotites which are very sensitive to weathering and probably covered the whole island. The oldest ferricrests on the top of lateritic profiles have been dated by paleomagnetism at late Oligocene[16]. Miocene sediments crop out in the area of Nepoui peninsulas and islets

where they form gently southwest-dipping layers (< 5°)[17-18] unconformably laying on the allochthonous ophiolitic units.

A. The Nepoui formations (Supplementary Figs. 4-6)

The Nepoui Group is divided into two formations[19]. The lower formation corresponds to c. 100 m thick reefal and lagoonal limestones that represent the earliest reefal post-obduction settlement. It is unconformably overlain by the 120 m thick upper formation. This last one starts with an 80 m thick, fluviatile to torrential cobble conglomerate, the components of which are weathered peridotites, serpentinites and basalts from the ophiolitic allochthonous substrate. Significant feature are the occurrences of pebbles of ferricrete and silcrete that reflect the erosion of older regolith. Silicified and ferruginous fossil woods fragments are common in the conglomerate[20], the deposition context of which is clearly terrestrial. This fluviatile and torrential unit grades upwards into a finer grained one where marine and terrestrial influences are equal. This intermediate unit, ca. 15 m thick, is made up of alternating bioclastic and lithoclastic sands, conglomerate lenses and calcareous mudstone. The plant and insect fossils have been found in these beds. They grade upward into a bioclastic limestone rich in coral, algae and echinid fragments, 25 m thick.

The age of the formations is Early Miocene, based on both biostratigraphy (benthic and planktic) and strontium isotope. The Lower Népoui Formation is Aquitanian. The Upper Népoui Formation, and therefore the fossiliferous beds described here, are Burdigalian.

Macrofossils from the Népoui Formation are nearly all three-dimensional molds of leaves filled with crystalline calcite. In some instances, this molding process preserved high quality details of leaf morphology while in others it did not. Remains of organic matter, including cuticle, are absent from all the fossils. The fossil flora in total (see Table S1) preserves 43 morphotypes, including one fern pinnule, one type of leafy conifer twig, two woody

infructescences, and 40 probable types of dicot leaves (35 of which are unequivocally distinct from each other). Morphotype determinations were based on differences in shapes, sizes, and most importantly, venation patterns. This allows us to distinguish morphotypes even with fragmentary and/or rare leaves. Most morphotypes are represented by only a few specimens (Supplementary Table 1). For 35 out of 40 morphotypes, the leaves were nonetheless clearly distinct from all others in their morphology and were considered unequivocal morphotypes. The other five morphotypes were considered likely but more equivocal (Table S1).

Taxonomic assignments within these broad groups are difficult due to variable preservation, the fragmentary nature of the fossils, the lack of cuticular anatomy, and the general difficulty of placing intertropical angiosperm leaf fossils. The conifer twig has small, imbricated scale leaves and most likely belongs to the Podocarpaceae, as similar kinds of scale-leafed Araucariaceae generally have larger diameter branches. But in the absence of cuticle anatomy, we cannot unambiguously assign them. The Népoui flora includes two types of woody infructescences, the larger of which we assigned to the genus *Gymnostoma* (Casuarinaceae) due to its large bracteoles that are widely separated by thick subtending bracts. We provisionally assign the smaller infructescence to *Gymnostoma* as well, because it also has relatively widely separated, highly protruding bracteoles, but it is not as well preserved and its morphology is more similar to that of other Casuarinaceae. We did not assign dicot leaf morphotypes to families.

Despite a lack of taxonomic resolution, dicot leaf physiognomy may be nonetheless useful to infer paleoclimate. In particular, we noted that the leaf areas of intact or largely intact specimens of the Népoui fossils are larger than those of extant species living in the sclerophyllous forest of the Pindai Peninsula today (Supplementary Fig. 6) and larger leaf size is often associated with higher precipitation. In order to more directly compare them, we quantified leaf area in 22 representative extant species sampled from the Pindai Peninsula flora;

these leaves were based on scans of herbarium specimens housed in the Paris Herbarium and identified by one of the authors (JM). For both fossil and extant leaves, total area was measured from digital photographs using Image J by tracing leaf areas. These data are preliminary; we plan to more thoroughly sample extant leaves for future work. Results are nevertheless consistent with our initial observation that leave are generally larger in the fossil assemblage. We also note that drip tips occur in a few of the morphotypes, further suggesting high precipitation.

B. The Fluvio-Lacustrine Formation (Figs. 3-4, Supplementary Figs. 7-9)

The Peridotite Nappe that resulted from late Eocene obduction in New Caledonia, is mainly formed of harzburgite and dunite, which are not overlain by oceanic crust, leaving ultramafic rocks exposed at the surface. New Caledonia emerged during or very soon after obduction at ~34 MY; and, after a period of vigorous uplift, an ultramafic regolith developed over peridotites and incidentally gave birth to supergene nickel ore. Although erosion of an older regolith has been already described in Early Miocene sediments[17], the fossil character of regolith development has been generally overlooked. However, the northwest coast of the island uplifted during the Early Miocene and erosion deeply eroded the Peridotite Nappe; as a result, it is now split in two sets. These are the “Massif du Sud” in the south (which includes the Mt Humboldt massif, formed of one single peridotite mass) and a series of isolated tectonic klippes spread along the west coast. At present, peridotites comprise approximately one third of island's surface. Based on paleomagnetic data, the age of the iron crust suggests that regolith in West Coast klippes developed before the latest Oligocene (~25 MY)[21], timing consistent with Coudray’s observations, and stopped at that time. By contrast, weathering probably continued in the rest of the ophiolite from Miocene to Recent[21].

The Fluvio-Lacustrine Formation refers to the sedimentary infill of depressions that are mainly located in the south of Massif du Sud; Yaté Basin, Plaine des Lacs, Rivière des Pirogues, and Creek Pernod (Fig. 1). This unit, with a maximum thickness of 70 m, comes from the erosion of weathering profiles developed over peridotites or gabbro cumulates[22]. Its base is locally formed of coarse conglomerate that contains boulders of peridotite and cuirasse. It is characteristically formed of fine-grained ferruginous material and serpentine, and displays diagenetic re-concentration of oxy-hydroxide located in discontinuities and within sedimentary beds. It also displays evidence for hydromorph (palustrine) pedogenesis with horizons of ferric crust, 'puppets' and plant roots encrusted with iron oxides, some of which are rich in fossil plant remains. In its major part, ferruginous cuirasses develop at its top (Plateau de Gertrude, Madeleine waterfall, etc.) in probable connection with an ancient water table. Border facies are generally formed of coarse sand to gravel-size cuirasse clasts with iron-rich silt matrix. Locally, these sediments are overlain by an alternation of fluvial coarse sands and clay. In most places, these sediments overlie *in situ* weathering profiles.

The morphology of endorheic basins in the southern Massif du Sud (Yaté, Plaines des lacs) has been interpreted by Trescases[23] as an intermediate stage in the development of karst landforms in ultramafic context, with very flat-bottomed wetland basins and a dominantly underground drainage which corresponds to the coalescence of sinkholes in poljes separated by residual rocky chains. However, some of the basins mentioned above do not meet this definition and have been clearly formed by sedimentary infill of an older drainage system.

A still pending problem is the age of these sediments; the works of Chardon & Chevillotte[24] and Chevillotte et al.[25] deal with the bulk of New Caledonia's post-obduction terrestrial deposits; they have explored the causes of regolith erosion, and proposed a long-term evolution mainly controlled by Oligocene-Miocene tectonics and global sea level change. Specifically, they compared the Early Miocene Nepoui Formation (NF, west coast)[17-18] and Goa N'Doro

Formation (GDF, Kouaoua village, east coast)[26]. For the latter, the authors postulated an Oligocene age based on the fact that the abandonment surface at the top of the GDF (surface 3 in [24]) is thought to be underneath the NF. Thereafter, the authors extrapolated the age of fluvial formations to the south of New-Caledonia based on this abandonment surface. Therefore, the age of the Fluvio-Lacustrine Formation would be Oligocene to Early Miocene. However, these correlations and the age deduced from them are contradicted by the discovery of Early Miocene coralgal limestone 120 m thick underneath Nepoui Formation. The age of these terrestrial sediments should be confirmed by independent methods (i.e. paleomagnetism, in progress). The Fluvio-Lacustrine Formation records a succession of events, which are, a first stage of torrential incision that eroded a pre-existent regolith and the underlying bedrock as well, followed by a second stage during which the resulting network is filled up in low energy conditions. The third phase consists of a stage of stability during which cuirasse developed and vegetation settled on the sedimentary infill. A final phase of incision led to the present morphology.

B.1 Terrestrial post-obduction sediments

The Fluvio-Lacustrine Formation, with a maximal thickness of 70 m, characteristically reworks weathering products of peridotites and gabbros (iron-rich laterites, kaolinitic clays)[22, 27-28]. Because of the lobed shape of some basins and the numerous dolines developed upon the infill, these basins were previously interpreted as 'poljes' based on doline coalescence[23]. However, the bedrock topography revealed by core drilling reveals an ancient river network controlled by rectilinear lineaments. In most basins, fluvial sediments rest directly upon *in situ* laterite, this feature means that erosion was lesser than in the north-western part of the island. The lack of well-defined outlets suggests partially endorheic evolution of these basins. In the Rivière des Pirogues Basin and Yaté Basin, sediments are limited to the northeast by the Unia-Goro uplifted block, which played the role of a dam allowing the accumulation of sediments.

The sedimentary succession often starts with a coarse torrential conglomerate that contains large boulders (up to 1 m) of peridotite and iron crust. This conglomerate is overlain by sands and silty-clays formed of fine-grained ferruginous material and serpentine. Hydromorphic (palustrine) pedogenesis is evidenced by ferruginous rhizocretions and duricrusts, some of which are rich in fossil plant remains. Diagenetic reconcentrations of iron oxihydroxide appear in discontinuities and within sedimentary beds[27-28]. In most basins, ferricretes develop on top of the sedimentary succession in probable connection with an ancient water table.

Indirect paleomagnetic dating of duricrusts and ferricrete developed within and on top of sediments respectively suggests formation during the Late Oligocene - Middle Miocene interval[27,29-30]. These ages are similar to that of autochthonous ferricretes of Tiébaghi (north) and Goro (south) but they are dominantly formed of reworked ultramafic regolith material, a fact which suggests that they were deposited and weathered again within a very short span of time. Brevity of this event and the absence of any other identified tectonic event during this interval suggest that it could be related to the same event that was responsible for the Miocene erosion and deposition of Népoui conglomerate. Younger ages obtained (10-20 MY and 0-5 MY) for *in situ* weathering profiles and fluvial sediments[27, 29-30] record continuous weathering, probably during two distinct episodes during the Miocene and Recent.

B.2 La Madeleine outcrop (Supplementary Figs. 7-8)

This outcrop is located near the northern boundary of the Plaine des Lacs Basin. The sedimentary succession is about six metres thick, dips 15° to the south-west, and rests upon *in situ* laterites. It begons with 1.8 m of heterometric conglomerate with sub-angular clasts of goethite and peridotite (from a few mm to 20-25 cm) included in a brown silty-sandy matrix with intercalated lenses of fine sand. This basal unit is overlain by 4.2 m of finer grain lenticular

to wavy bedded sediments (coarse and fine fraction in equivalent proportions). The coarse fraction consists in heterometric, sub-angular gray to black gravel and granule lenses (3 mm to 4 cm thick) and angular blocks of collapsed laterite wherein chromite grains and serpentine vein clasts remain. It is overlain by 20 cm of sub-rounded and poorly-sorted goethite granules and gravels, and then by 30 cm of coarsening upwardsands and ferruginous granules. Next 25 cm of dark-brown silt and clay yield a ferruginous duricrust (4 cm thick), which contains well-preserved fossil-leaves. Finally, a pisolithic/nodular ferricrete, 40 cm thick forms the top of the succession.

B.3. Pont des Japonais outcrop (Supplementary Fig. 9)

This section starts with 2 m of alternating blue-green and purple-red fine-grain sands overlain by 5 m of silt and clay. In the first meter, the grain size diminishes from purple-red sands to wine-colored silts. Then, in the next 50 cm, most sediment is wine-colored and more or less hardened. White silica is found impregnating porosity as meniscus-shaped lens or white coating. In the next 3.5 m, the colour changes progressively into ochre. Manganese oxide stains and ferruginous duricrusts parallel to stratification are found in the infill, as well as some carbonaceous wood clasts (max 0.5 cm) observed at -2 m (Fig. 4).

At 0 m, a silt/clay bed, ≈ 15 cm thick, indurated by iron oxi-hydroxide, yielded an impressive amount of fossil leaves. 1.5 m above this level, the grain size increases dramatically to form a micro-conglomerate with peridotite and gabbro pebbles and supergene silica clasts mixed in a matrix of coarse sand. The whole set has been subsequently lateritized. In the last 2.5 m the grain size decreases again to ochre to brown silts and clays in which sedimentary structures have been erased during weathering. Several levels of ferruginous rhizocretions signal episodes of vegetation settlement.

Extended information on flora and fauna

Beetle elytra from 'La Madeleine' outcrop

Coleoptera Scarabaeidae Dynastinae, *Hemicyrthus* species

Two elytra, joined, apparently fused; outer margins distinctly rounded; elytra glabrous and smooth, without ridges, but with 4–6 rudimentary striae, mainly on the anterior half; punctures fine and dense, in posterior half transversally wrinkled. Micropunctures sometimes shallow, but visible; width of a elytra 9 mm, length of preserved part 18 mm

This type of elytra can be found in Neo-Caledonian Scarabaeidae: Dynastinae. Among them, only the genus *Hemicyrthus* has the same shape of elytra[31]. More precisely, the fossil strongly resembles the elytra of the recently described *Hemicyrthus blaffarti* Krell & Theuerkauf, 2015 from the locality Rivière Bleue. This locality is very close to the Saut de la Madeleine. This modern species has the same ornamentation of the elytra and differs from the fossil in smaller width of joined elytra, ca. 15 mm instead of 18 mm[32]. Thus our fossil probably corresponds to an extinct species of *Hemicyrthus*, very close to *H. blaffarti*.

Current knowledge on the fossil plant records from New Caledonia

The current knowledge on fossil plants from New Caledonia is very scarce. Salard and Avias[33] revised the knowledge on the fossil plants found in New Caledonia. They cited the presence of silicified woods and imprints of leaves from the Triassic (Carnian) of Teremba-Mara near Mouindou (Western Coast). They also indicated silicified woods in the Lower Jurassic of 'Baie Inaccessible'; in the Middle Jurassic of the Western coast of the island. They also indicated the presence of wood fragments and leaves in the Upper Cretaceous of the 'Portes de Fer', near Nouméa, and near Mouindou. Vozenin-Serra and Salard-Cheboldaeff[34] described three new species of Permo-Triassic fossil wood. Salard-Cheboldaeff et al.[35] added fossil woods:from

Tiebaghi: Cenozoic (post-Eocene) woods; Poya: Cenozoic (post-Eocene) palm stipa; Voh: araucarian wood.

Table of previous fossil localities with continental fossil plants in New Caledonia

**Locality**	**Age/period**	**Fossils**	**Reference**
Moindou	Triassic	wood, leaves	33
Baie inaccessible	Middle Jurassic	silicified wood	33
Noumea	Upper Cretaceous		33
?	Permo-Triassic	fossil wood	34
Tiebaghi	post-Eocene	fossil wood (5 taxa)	35
Poya	post-Eocene	palm stipa	35
Voh	?	Araucarian wood	35

Also Lacroix[36] indicated the presence of a fossil beetle (Tenebrionidae: *Adelium externecostatum* Bates, 1873, extant species) fossilized in Garnierite.

References

1. Schellart, W. P. A late Cretaceous and Cenozoic reconstruction of the southwest pacific region: tectonic controlled by subduction and slab rollback process. *Earth Science Reviews* **76**, 191–233 (2006).
2. Crawford, A. J. et al. 120 to 0 MY tectonic evolution of the southwest Pacific and analogous geological evolution of the 600 to 220 MY Tasman Fold Belt system. *Special Paper of the Geological Society of America* **372**, 383–403 (2003).

3. Cluzel, D. et al. Tectonic accretion and underplating of mafic terranes in the Late Eocene intraoceanic fore-arc of New Caledonia (Southwest Pacific) geodynamic implications. *Tectonophysics* **340**, 23–59 (2001).
4. Tissot, B. & Noesmoen, A. Les bassins de Nouméa et de Bourail (Nouvelle-Calédonie). *Revue de l'Institut Français du Pétrole* **13**, 739–759 (1958).
5. Aitchison, J. C. et al. Eocene arc-continent collision in New Caledonia and implications for regional southwest Pacific tectonic evolution. *Geology* **23**, 161–164 (1995).
6. Moore, P. R. Stratigraphy, composition, and environment of deposition of the Whangai Formation and associated Late Cretaceous - Paleocene rocks, eastern North Island, New Zealand. *New Zealand Geological Survey Bulletin* **100**, 1–82 (1988).
7. Cooper, R. A. (ed.) The New Zealand Geological Timescale. *Institute of Geological & Nuclear Sciences Monograph* **22**, 1–284 (2004).
8. Maurizot, P. First sedimentary record of the pre-obduction convergence in New Caledonia: formation of an Early Eocene accretionary complex in the north of Grande Terre and emplacement of the 'Montagnes Blanches' nappe. *Bulletin de la Société Géologique de France* **182**, 479–491 (2011).
9. Nicholson, K. N. et al. Geochemistry and age of the Nouméa Basin lavas, New Caledonia: Evidence for Cretaceous subduction beneath the eastern Gondwana margin. *Lithos* **125**, 659–674 (2011).
10. Orton, K. T. Constraining the age of the Nouméa Basin: Isotope age and paleomagnetic data from New Caledonia. Master of Science in Geology, Ball State University, Muncie, Indiana. (2012).
11. Cluzel, D. et al. Detrital zircon records of Late Cretaceous syn-rift sedimentary sequences of New Caledonia: an Australian provenance questioned. *Tectonophysics* **501**, 17–27 (2011).

12. Cluzel, D. et al. An outline of the Geology of New Caledonia; from Permian–Mesozoic Southeast Gondwanaland active margin to Cenozoic obduction and supergene Evolution. *Episodes* **35**, 72–86 (2012).
13. Cluzel, D. et al. Discovery of Early Cretaceous rocks in New Caledonia (Southwest Pacific). New geochemical and U-Pb zircon age constraints on the transition from subduction to marginal breakup. *The Journal of Geology* **118**, 381–397 (2010).
14. Aronson, J. L. & Tilton, G. R. Probable Precambrian detrital zircons in New Caledonia and Southwest Pacific continental structure. *Geological Society of America Bulletin* **82**, 3449–3456 (1971).
15. Lagabrielle, Y. et al. Post-Eocene extensional tectonics in Southern New Caledonia (SW Pacific): Insights from onshore fault analysis and offshore seismic data. *Tectonophysics* **403**, 1–28 (2005).
16. Sevin, B. et alFirst paleomagnetic dating of ferricrete in New Caledonia: new insight on the morphogenesis and paleoweathering of 'Grande Terre'. *Terra Nova* **24**, 77–85 (2011).
17. Coudray, J. Recherches sur le Néogène et le Quaternaire marins de la Nouvelle-Calédonie; contribution de l'étude sédimentologique à la connaissance de l'histoire géologique post-éocène, in Expédition française sur les récifs coralliens de la Nouvelle-Calédonie; volume Huitième. Thèse Doctorat d'Etat, Montpellier. (1976).
18. Paris, J. P. Géologie de la Nouvelle-Calédonie. Un essai de synthèse. *Mémoires du Bureau de Recherches Géologiques et Mi*nières **113**, 1–278 (1981).
19. Maurizot, P. et al. Post-obduction carbonate system development in New Caledonia (Népoui, Lower Miocene). *Sedimentary Geology* **331**, 42–62 (2016).

20. Genise, J. F. et al. *Asthenopodichnium* in fossil wood: different trace makers as indicators of different terrestrial palaeoenvironments. *Palaeogeography, Palaeoclimatology, Palaeoecology* **365-366**, 184–191 (2012).

21. Sevin, B. et al. First paleomagnetic dating of ferricrete in New Caledonia: new insight on the morphogenesis and paleoweathering of 'Grande Terre'. *Terra Nova* **24**, 77–85 (2012).

22. Guillon, J. H. et al. Carte géologique à l'échelle du 1/50000. Notice explicative sur la feuille Prony. Bureau de Recherches Géologiques et Minières, Paris, 1–38 (1972).

23. Trescases, J. J. L'évolution géochimique supergène des roches ultrabasiques en zone tropicale; formation des gisements nickelifères de Nouvelle-Calédonie. *Mémoires ORSTOM* **78**, i–xix + 1–258 (1975).

24. Chardon, D. and Chevillotte, V. Morphotectonic evolution of the New Caledonia ridge (Pacific Southwest) from post-obduction tectono-sedimentary record. *Tectonophysics* **420**, 473–491 (2006).

25. Chevillotte, V et al. Long-term tropical morphogenesis of New Caledonia (Southwest Pacific): importance of positive epeirogeny and climate change. *Geomorphology* **81**, 361–375 (2006).

26. Orloff, O. & Gonord, H. Note préliminaire sur un nouveau complexe sédimentaire continental situé sur les massifs du Goa N'Doro et de Kadjitra (régions côtières à l'est de la Nouvelle-Calédonie). *Comptes-Rendus de l'Académie des Sciences*, Paris, (D) **267**, 5–8 (1968).

27. Folcher, N. Contrôles géodynamique et climatique du système fluvio-lacustre de Nouvelle-Calédonie, conséquences sur les gisements de nickel latériques. Doctoral Thesis, UNC, Nouméa, New-Caledonia. (2016).

28. Folcher, N. et al. Neogene terrestrial sediments: a record of the post-obduction history of New Caledonia. *Australian Journal of Earth Sciences: An International Geoscience Journal of the Geological Society of Australia* **62**, 479–492 (2015).

29. Quesnel, F. et al. Appui scientifique à la cartographie du régolithe (Grande Terre de Nouvelle-Calédonie). Rapport intermédiaire, phase 1 (2007-2008). BRGM/RP-56650-FR. (2008).

30. Ricordel-Prognon, C. et al. Appui scientifique à la cartographie du régolithe (Grande-Terre de Nouvelle-Calédonie). Rapport intermédiaire, phase 3. BRGM/RP-60159-FR. (2011).

31. Paulian, R. Les Coléoptères Scarabaeoidea de Nouvelle-Calédonie. Editions de l'ORSTOM, *Collection Faune Tropicale*, Paris **29**, 1–164 (1991).

32. Krell, F.-T. & Theuerkauf, J. A new species of the endemic genus *Hemicyrthus* Reiche (Coleoptera: Scarabaeidae: Dynastinae) from New Caledonia, with a revised key. *Zootaxa* **4048**, 281–290 (2015).

33. Salard, M. & Avias, J. Contribution à la connaissance de la flore fossile de la Nouvelle Calédonie, avec une introduction stratigraphique. *Palaeontographica* (B) **124**, 1–44 (1968).

34. Vozenin-Serra, C. & Salard-Cheboldaeff, M. Les bois minéralisés permo-triasiques de Nouvelle-Calédonie. Implications phylogénétique et paléogéographique. *Palaeontographica* (B) **225**, 1–25 (1992).

35. Lacroix, A. Sur une pseudomorphose d'insecte en nouméite (Nouvelle-Calédonie). *Bulletin de la Société Française de Minéralogie* **26**, 303 (1903).

36. Salard-Cheboldaeff, M. et al. Bois minéralisés cénozoïques de Nouvelle-Calédonie. *Palaeontographica* (B) **288**, 65–97 (2012).

37. Jaffré, T. et al. Changes in the vegetation of New Caledonia Pacific Ocean during the Tertiary: the vegetation and flora on ultramafic rocks. *Bulletin du Muséum National d'Histoire Naturelle* (B *Adansonia*) **9**, 365–392 (1987).

38. Pillon, Y. Time and tempo of diversification in the flora of New Caledonia. *Botanical Journal of the Linnean Society* **170**, 288–298 (2012).

Supplementary figures for SI (1 to 8)

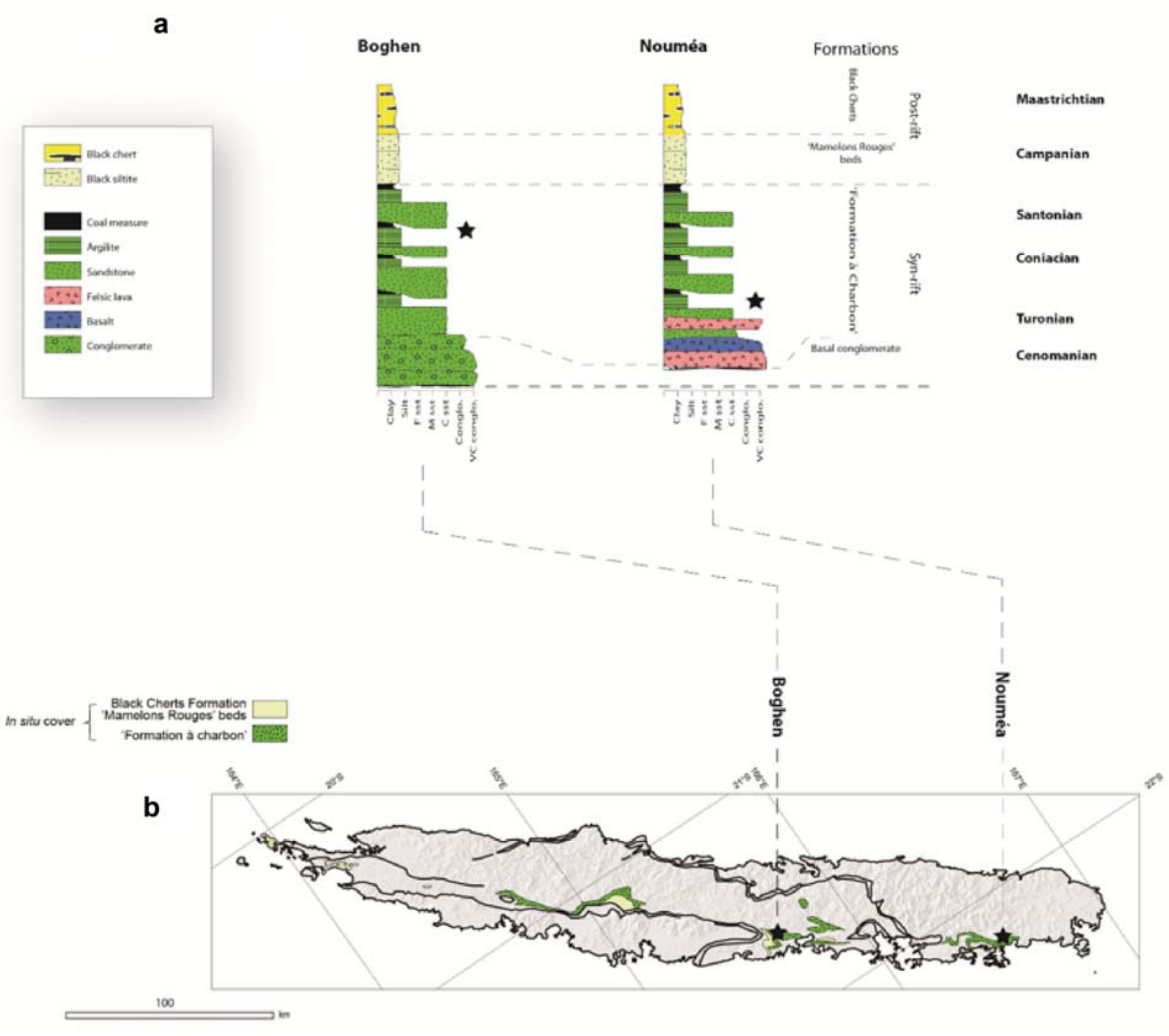

Supplementary Figure 1 | Cretaceous sites. Haute Nessadiou (Boghen) and Haut Robinson (Noumea), locations and logs. (**a**) representative logs through the 'Charbon' Formation in Noumea and Boghen with age correlations; (**b**) map of New Caledonia showing the distribution of the 'Charbon' Formation. Copyright P. Maurizot.

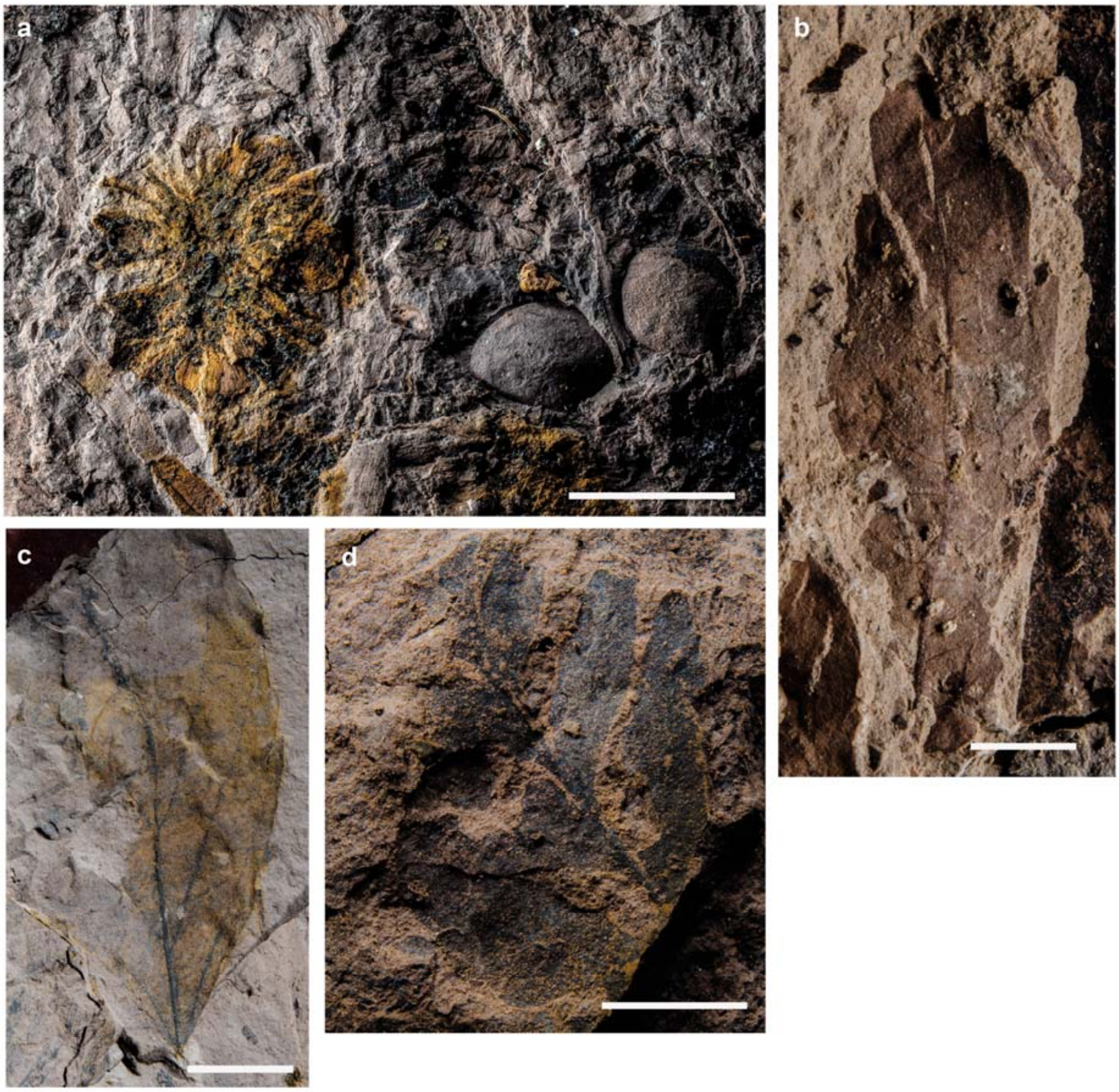

Supplementary Figure 2 | Fossil plants from Haute Nessaoui (Upper Cretaceous). (**a**) taxodiaceous cone similar to *Austrosequoia* and large isolated seeds; (**b, c**) isolated angiosperm leaves; (**d**) dissected leaf or fern frond. Coll. SGNC: New Caledonia Geological Survey. Copyright R. Garrouste. Scale bars = 1cm.

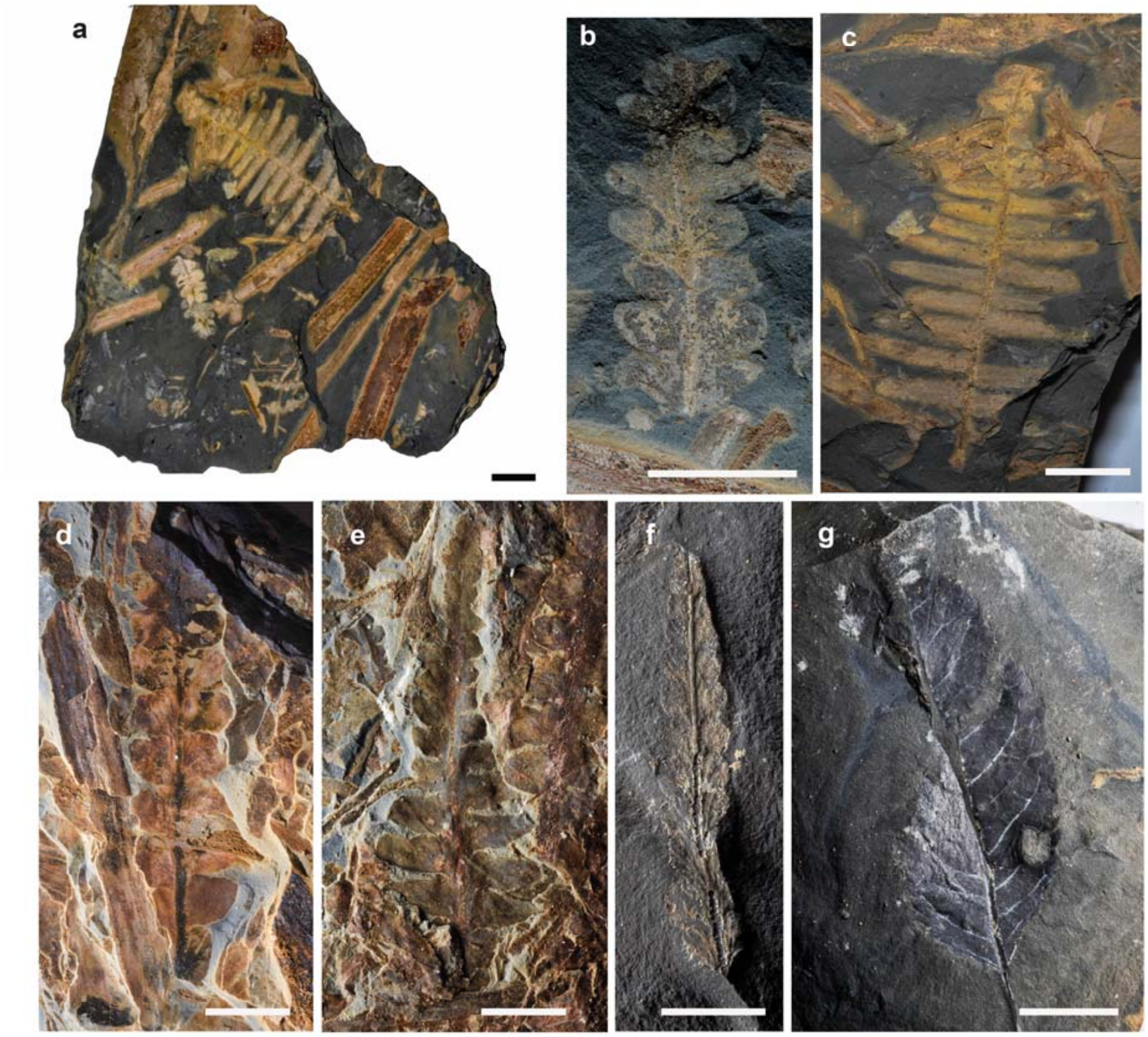

Supplementary Figure 3 | Fossil plants from the Haut Robinson outcrop. (**a**) slab with comminuted plant material on bedding plane comprising stems and fragments of fern fronds; (**b-f**) fern pinnae and pinnules: (**b, d, f**) ?*Microphyllopteris* sp., (**c, e**) terminal pinnae of an unidentified fern; (**g**) small angiosperm leaf with strongly serrate and toothed margin. Copyright R. Garrouste. Scale bars = 1cm.

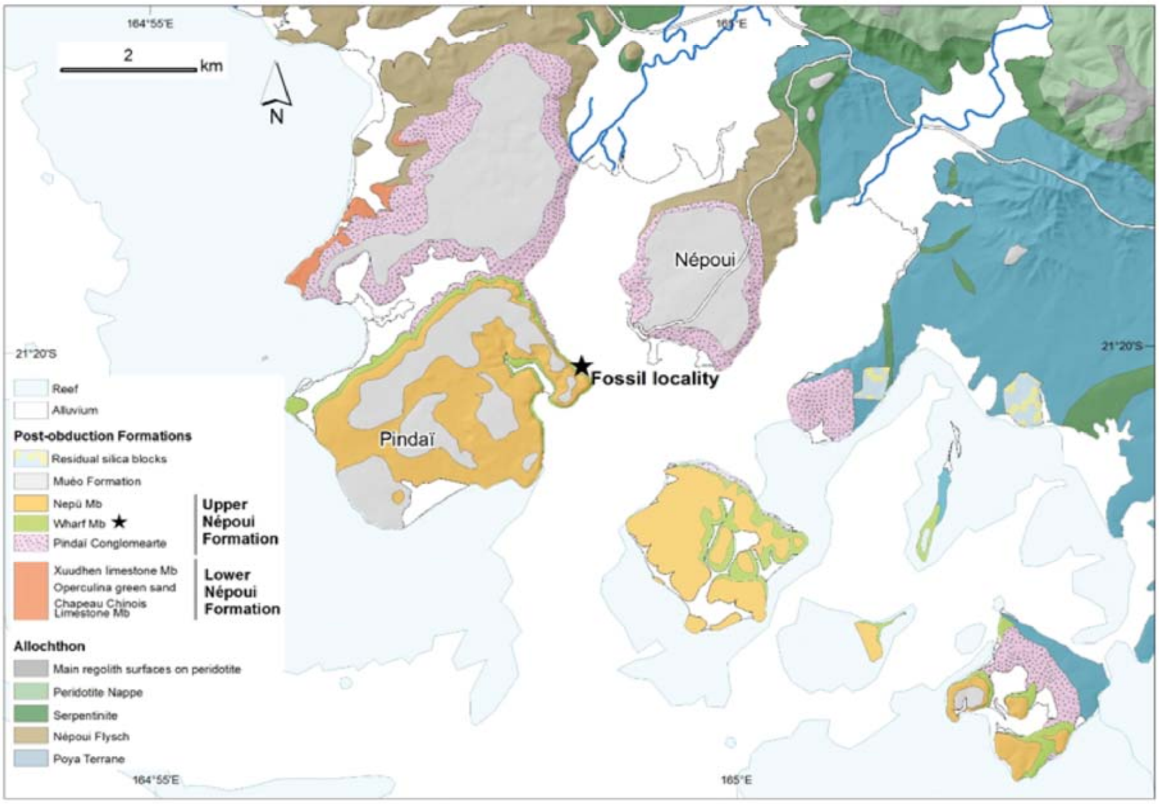

Supplementary Figure 4 | Geological map of Nepoui area. Position of the new Miocene fossil outcrop. Copyright P. Maurizot.

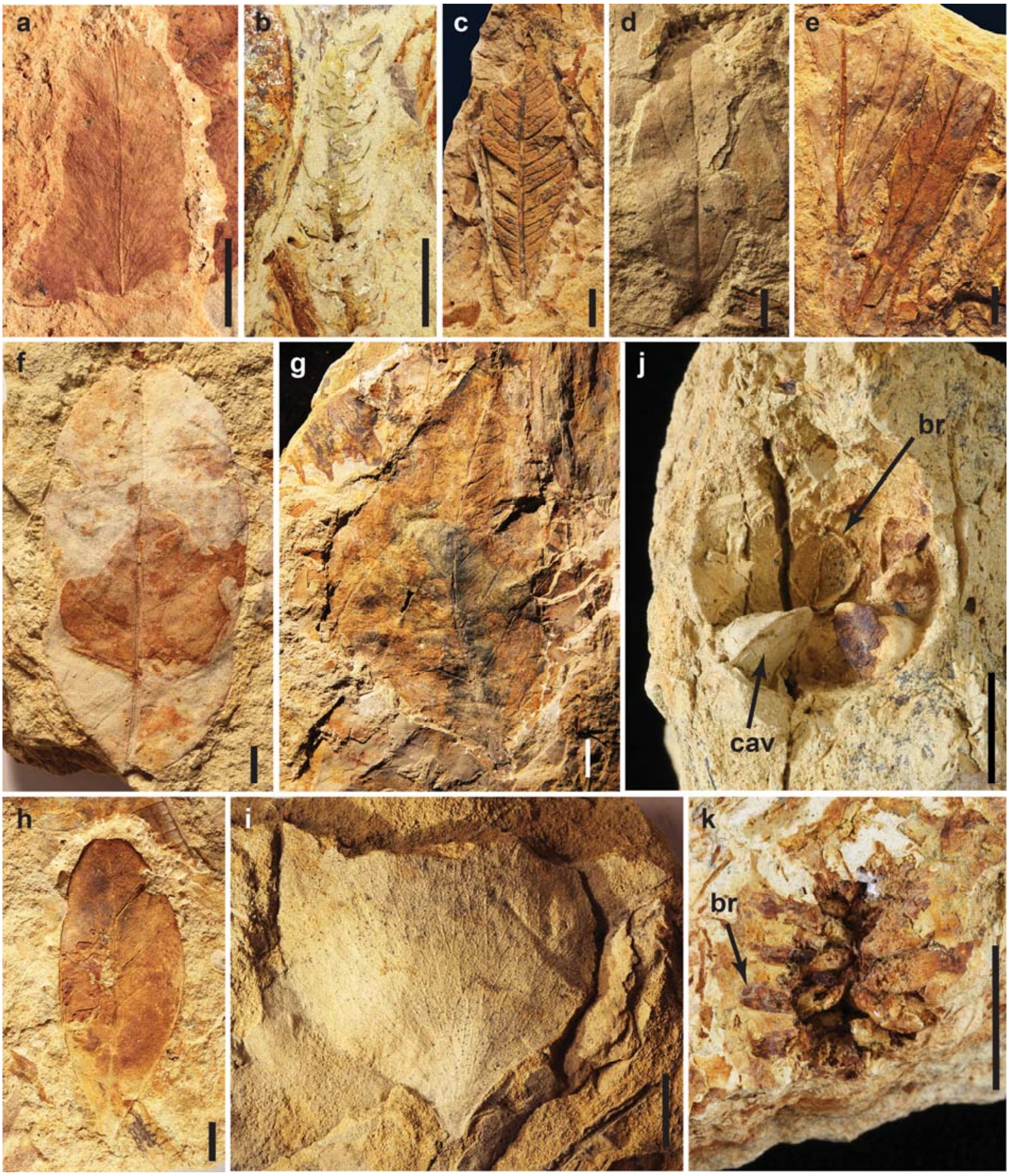

Supplementary Figure 5 | Fossil plants from Nepouí, Pindai Peninsula. (**a**) fern pinnule; (**b**) conifer branch with imbricate scale-leaf foliage; (**c-i**) angiosperm leaves of mostly uncertain affinities; (**j-k**) *Gymnostoma* infructescence. br bracteoles, cav cavity. Copyright A. Leslie. Scale bars = 1 cm.

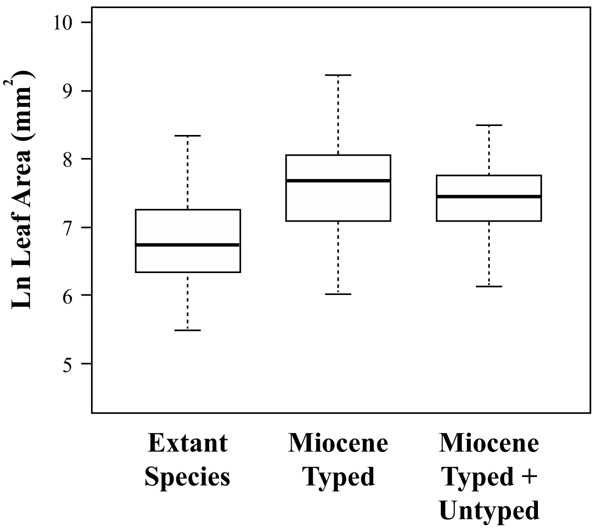

Supplementary Figure 6 | Comparison of leaf areas of extant Nepoui species with fossil leaves. Fossils include those that were typed, as well as typed plus other leaves that could not be typed due to poor preservation.

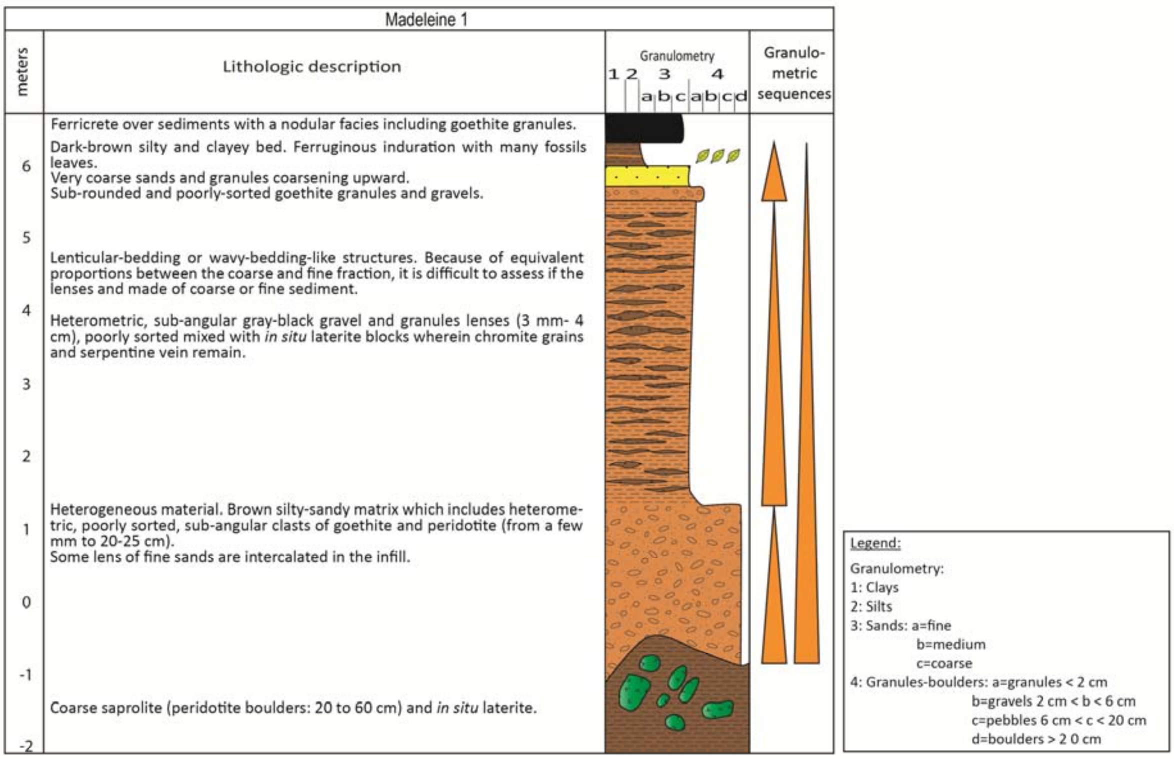

Supplementary Figure 7 | Geological log of La Madeleine locality. Fluvio-Lacustrine Formation of Middle(?) Miocene age. Copyright P. Maurizot.

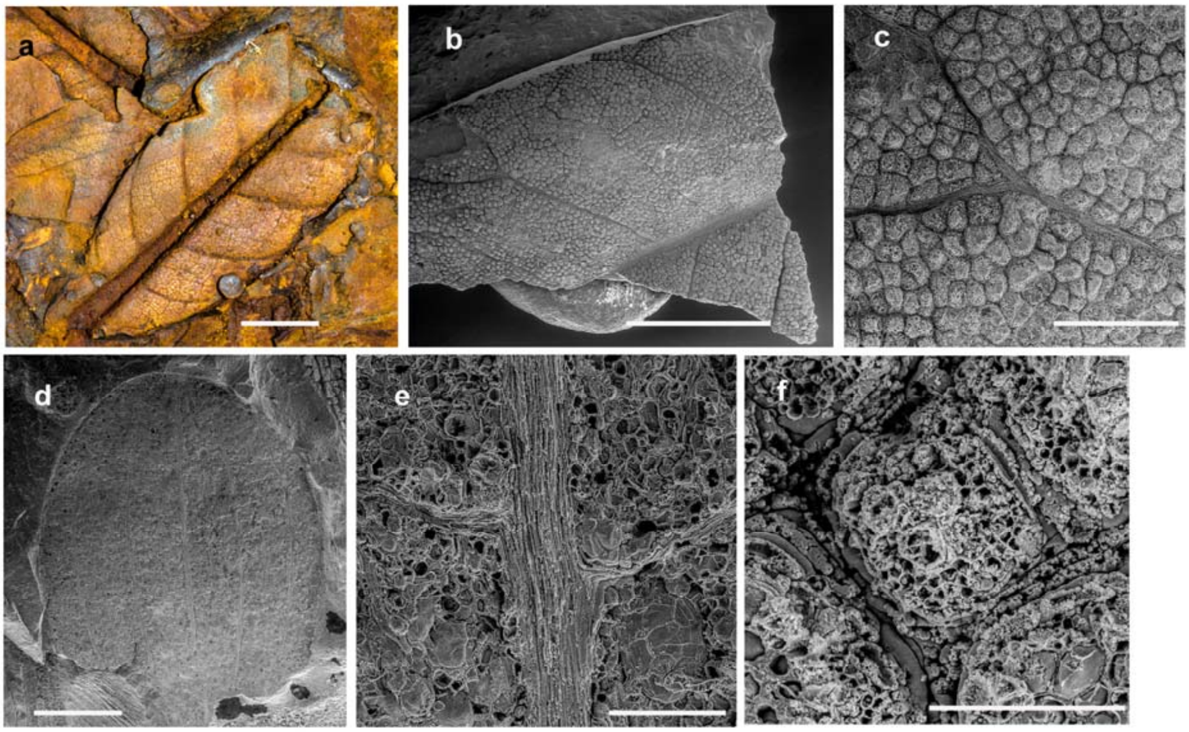

Supplementary Figure 8 | Fossil leaves from the La Madeleine outcrop. Ferricrust within the Fluvio-Lacustrine Formation, Middle(?) Miocene. (**a**) example of fossil leaf in hand specimen with details of leaf venation; (**b-f**) environmental SEM images of fine detail preserved by iron oxides detected using EDS element analysis; (**b**) overview of leaf surface illustrating venation pattern; (**c**) detail of areolation; (**d**) small leaf or bract with veins and cells; (**e-f**) close up of cells over vein. Copyright R. Garrouste. Scale bars a = 1 cm; b = 3 mm; 1 = 1 mm; d = 100 µm; e = 2mm; f = 200 µm.

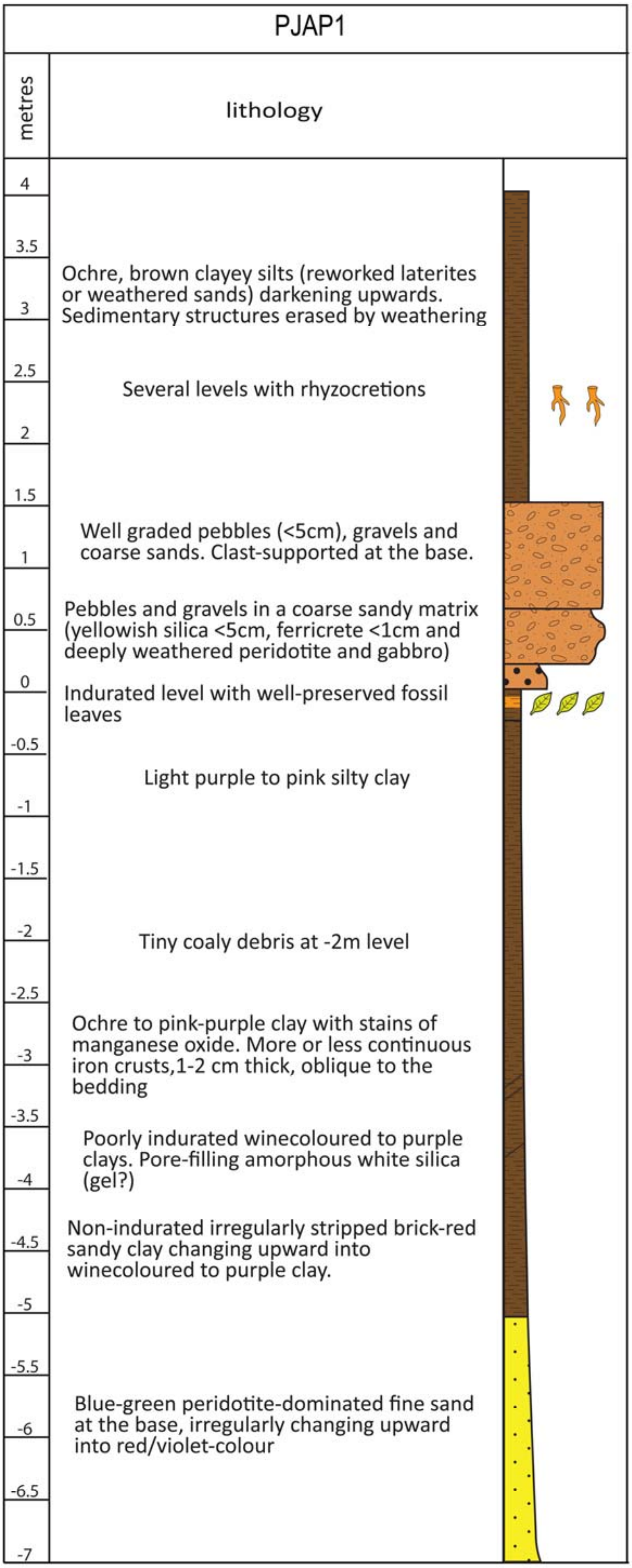
PJAP1
metres
lithology
4
3.5
3
2.5
2
1.5
1
0.5
0
-0.5
-1
-1.5
-2
-2.5
-3
-3.5
-4
-4.5
-5
-5.5
-6
-6.5
-7
Ochre, brown clayey silts (reworked laterites or weathered sands) darkening upwards. Sedimentary structures erased by weathering
Several levels with rhyzocretions
Well graded pebbles (<5cm), gravels and coarse sands. Clast-supported at the base.
Pebbles and gravels in a coarse sandy matrix (yellowish silica <5cm, ferricrete <1cm and deeply weathered peridotite and gabbro)
Indurated level with well-preserved fossil leaves
Light purple to pink silty clay
Tiny coaly debris at -2m level
Ochre to pink-purple clay with stains of manganese oxide. More or less continuous iron crusts,1-2 cm thick, oblique to the bedding
Poorly indurated winecoloured to purple clays. Pore-filling amorphous white silica (gel?)
Non-indurated irregularly stripped brick-red sandy clay changing upward into winecoloured to purple clay.
Blue-green peridotite-dominated fine sand at the base, irregularly changing upward into red/violet-colour

Supplementary Figure 9 | Geological log of Pont des Japonais locality. Fluvio-Lacustrine Formation of Middle(?) Miocene age. Copyright P. Maurizot.

Group	Morphotype	Specimens	Organ type	Affinities
Ferns	7	2	pinnule	Filicales
Conifers	43	3	leafy twig	cf. Podocarpaceae
Angiosperm	41	2	infr.	cf. *Gymnostoma*
	42	1	infr.	*Gymnostoma*
	1	6	leaf	unidentified
	2	2	leaf	unidentified
	3	2	leaf	unidentified
	4	14	leaf	unidentified
	5	1	leaf	unidentified
	6	2	leaf	unidentified
	7	1	leaf	unidentified
	8	1	leaf	unidentified
	9	1	leaf	unidentified
	10	1	leaf	unidentified
	11	1	leaf	unidentified
	12	1	leaf	unidentified
	13	1	leaf	unidentified
	14*	1	leaf	unidentified
	15	1	leaf	unidentified
	16	1	leaf	unidentified
	17	1	leaf	unidentified
	18	1	leaf	unidentified
	19	2	leaf	unidentified
	20	1	leaf	unidentified
	21*	1	leaf	unidentified
	22	1	leaf	unidentified
	23	1	leaf	unidentified
	24	2	leaf	unidentified
	25	1	leaf	unidentified
	26*	1	leaf	unidentified
	27	2	leaf	unidentified
	28	1	leaf	unidentified
	29	1	leaf	unidentified
	30	2	leaf	unidentified
	31	1	leaf	unidentified
	32*	1	leaf	unidentified
	33	1	leaf	unidentified
	34	1	leaf	unidentified
	35	1	leaf	unidentified

	36	1	leaf	unidentified
	37	1	leaf	unidentified
	38	3	leaf	unidentified
	39	1	leaf	unidentified
	40*	1	leaf	unidentified

Supplementary Table 1 | List of morphotypes and their abundance in the Népoui Formation. Morphotypes denoted by an asterisk are probable types, but whose preservation precludes unequivocal assignment as distinct types; infr.: infructescence

Supplementary Table 2 | Comparison between Middle(?) Miocene and Extant plant morphotypes (La Madeleine and Pont des Japonais outcrops). Number of examined specimens; types of fossils; taxonomic attributions; characters used for determinations (see excel document)

	H-Rob	H-Ness	Nepoui	Mad	Pjap	Ext
Gleicheniaceae	1	0	0	0	0	1
Osmundaceae	1	0	0	0	0	1
Dicksoniaceae	1	0	0	0	0	1
Podocarpaceae	1	0	1	1	0	2
Araucariaceae	1	2	0	1	0	3
Cupressaceae	0	2	0	0	0	1
Cycadales	0	1	0	0	0	1
Angiosperm undet.	1	1	3	3	3	3
Casuarinaceae	0	0	2	0	0	2
Epidacridaceae	0	0	0	1	1	2
Myrtaceae	0	0	0	1	0	1
Malvaceae	0	0	0	1	1	1
Calophyllaceae	0	0	0	0	1	1
Lauraceae	0	0	0	0	1	1
Rhamnaceae	0	0	0	0	1	1
Filicales	1	0	1	0	0	1
Thymelaeaceae	0	0	0	1	0	1

Supplementary Table 3 | Plant family distribution in the different outcrops

Supplementary Data | Comparison between extant and Middle(?) Miocene plant morphotypes (La Madeleine and Pont des Japonais outcrops)
